# CAPE activates AMPK and Foxo3 signaling to induce growth inhibition and ferroptosis in triple-negative breast cancer

**DOI:** 10.1371/journal.pone.0315037

**Published:** 2024-12-27

**Authors:** Qilu Fang, Qichuan Fang, Rui Cheng, Tingting Feng, Wenxiu Xin

**Affiliations:** 1 Hangzhou Institute of Medicine (HIM), Zhejiang Cancer Hospital, Zhejiang, Hangzhou, China; 2 School of Medical Technology and Information Engineering, Zhejiang Chinese Medical University, Zhejiang, Hangzhou, China; 3 School of Pharmacy, Nanchang University, Jiangxi, Nanchang, China; The Second Affiliated Hospital of Guangzhou Medical University, CHINA

## Abstract

**Purpose:**

Approximately 20% of all breast cancer cases are classified as triple-negative breast cancer (TNBC), which represents the most challenging subtype due to its poor prognosis and high metastatic rate. Caffeic acid phenethyl ester (CAPE), the main component extracted from propolis, has been reported to exhibit anticancer activity across various tumor cell types. This study aimed to investigate the effects and mechanisms of CAPE on TNBC.

**Methods:**

MDA-MB-231 and MDA-MB-468 cells were treated with CAPE. CCK8 and colony formation assays were performed to analyze cell proliferation. Western blot, TUNEL and Annexin V-FITC/PI staining methods were employed to assess cell apoptosis. ROS, MDA, SOD, GSH, C11-bodipy staining, along with measurements of GPX4 and Ferritin levels, were utilized for ferroptosis detection. Western blot and immunofluorescence analysis were used to assess key regulatory molecules. The cells were subjected to treatments involving ferroptosis inhibition, AMPK inhibition, or Foxo3 inhibition, followed by CAPE administration to assess cell proliferation, apoptosis, and ferroptosis. Tumor xenografts were used to evaluate the antitumor efficacy of CAPE.

**Results:**

CAPE not only suppressed cell proliferation but also promoted apoptosis followed by ferroptosis. Co-incubation with Fer-1 (a ferroptosis inhibitor) diminished CAPE’s suppressive effects on proliferation and apoptosis induction. CAPE treatment enhanced the phosphorylation of AMPK and promoted the nuclear translocation of Foxo3. Inhibition of both AMPK and Foxo3 by siRNAs or inhibitors (Compc, TIC10) reversed the growth retardation induced by CAPE as well as its pro-apoptotic effects leading to ferroptosis. Specifically, AMPK inhibition abrogated the CAPE-induced nuclear translocation of Foxo3. CAPE significantly inhibited tumor growth in nude mice bearing TNBC xenografts.

**Conclusion:**

CAPE possesses a resistance effect on TNBC via activation of AMPK and Foxo3 signaling pathways.

## Introduction

Breast cancer (BC) is one of the most prevalent malignancies and remains the leading cause of cancer-related deaths among women, despite significant advances in diagnosis and therapeutic methods [1, 2]. Among BC subtypes, triple-negative breast cancer (TNBC), characterized by the absence or low expression of estrogen receptor (ER), progesterone receptor (PR), and human epidermal growth factor receptor 2 (HER2) [3, 4], poses the greatest challenge due to its aggressive nature, high metastatic potential, and poor prognosis [5, 6]. Currently, chemotherapy serves as the primary treatment option for TNBC, and can to some extent prolong the survival of patients. However, the side effects and toxicities associated with chemotherapy reduce the quality of life both physically and psychologically. Furthermore, chemotherapy resistance is a major difficulty encountered in the treatment process and results in tumor progression [7, 8]. There is an urgent need to identify novel therapeutic agents that can effectively inhibit tumor progression.

In recent years, research into natural products and Chinese herbal medicines as potential sources of anti-tumor agents has gained considerable momentum. Caffeic acid phenethyl ester (CAPE), a major component of propolis, has garnered significant interest due to its pharmacological activities, including anti-inflammatory, anti-oxidant, and anti-cancer effects [9]. Previous studies have demonstrated that CAPE exhibits chemopreventive propertiesby inhibiting cell proliferation and inducing apoptosis in various cancer cell lines [10–12].

Ferroptosis, a distinct type of cell death, is characterized by the accumulation of mitochondrial dysfunction and iron-dependent lipid reactive oxygen species (Lip-ROS). This process can be triggered by various mechanisms [13, 14]. A crucial regulator of ferroptosis is the GXP4-GSH system. Specifically, the enzyme GPX4 facilitates the clearance of lipid peroxides while glutathione (GSH) aids GPX4 in catalyzing these peroxides. The GSH-dependent GPX4 is inactivated when GSH synthesis is inhibited, leading to an accumulation of Lip-ROS within the cell and ultimately oxidative damage to the cell and ferroptosis [15, 16]. Research has indicated that TNBC cells are susceptible to ferroptosis [17]. The therapeutic effect of ferroptosis agonists in treating tumors can be significantly enhanced when administered in combination. Nevertheless, ferroptosis agonists possess double-edged toxicity, potentially harming normal cells and organs [18, 19].

AMP-activated kinase (AMPK), an essential energy sensor, governs the cellular energy homeostasis. AMPK becomes activated in response to energy deprivation, subsequently stimulating ATP generation and modulating metabolic energy to sustain cellular function [20]. AMPK has not only attracted attention as a therapeutic target for obesity and metabolic disorders but has also emerged as a promising metabolic tumor suppressor, playing an essential role in cancer prevention and treatment [21, 22]. Studies have shown that metformin, an AMPK activator, can reduce cancer incidence and hinder its progression [23]. AMPK activation exerts tumor-suppressive effects by modulating metabolism, inhibiting cell proliferation, and inducing apoptosis, including ferroptosis. An increasing number of studies underscores the critical tumor suppressor function of AMPK in various cancers.

Forkhead box O3 (Foxo3), also known as Foxo3a and FKHR-L1, is recognized as a critical tumor suppressor. This transcription factor possesses the unique ability to shuttle between the cytoplasm and nucleus, where it regulates target gene transcription by binding to specific DNA sequences [24]. As a member of the Foxo subfamily, Foxo3 oversees various cellular functions, including cell proliferation, cell cycle progression, DNA damage response, apoptosis, and tumorigenesis. The inactivation or dysregulation of Foxo3 has been implicated in the pathogenesis of various cancers, including breast, liver, colon, prostate, bladder, and nasopharyngeal cancers [25, 26]. Recent research highlights the potential of Foxo3 as a therapeutic target in cancer management, underscoring its role as a tumor suppressor.

In this study, we examined the effect and underlying mechanism of CAPE on TBNC, with the following objectives: (1) to elucidate the inhibitory effect of CAPE on cell proliferation and its stimulatory effect on apoptosis in TNBC cells, (2) to investigate the potential mechanisms by which CAPE acts in TNBC cells, specifically its influence on ferroptosis and the AMPK and Foxo3 signaling pathways, (3) to investigate the contribution of AMPK and Foxo3 to the anti-tumor activity of CAPE, and (4) to validate the anti-tumor efficacy and mechanism of CAPE in vivo.

## Materials and methods

### Cell culture

The human TBNC cell line MDA-MB-231 and MDA-MB-468 were obtained from the Shanghai Institute of Biochemistry and Cell Biology, and were maintained in RPMI-1640 medium (Gibco, Thermo Fisher Scientific) supplemented with 10% fetal bovine serum (FBS), 100 U/mL of penicillin, and 100 mg/mL of streptomycin. Cells were cultured at 37°C in a humidified incubator with 95% air and 5% CO_2_.

### Cell viability assay

The CCK8 assay was used to determine cell viability. MDA-MB-231 and MDA-MB-468 cells (5 × 10^3^ cells/well) were seeded in 96-well plates and incubated for 24 h. Subsequently, the cells were transfected with siRNAs or treated with CAPE (Selleck), Fer-1 (Selleck), Compound C (Compc, Selleck), or TIC10 (Selleck) as specified in individual experiments. After the treatment, the cell medium was removed, CCK8 reagent (1:10, cat. no. C0037, Beyotime Biotechnology) was added, and the cells were incubated at 37°C for 30 min. Absorbance was measured at 595 nm using an ELISA microplate reader.

### Cell colony formation assay

MDA-MB-231 and MDA-MB-468 cells (5 × 10^4^ cells/well) were seeded in 6-well plates and incubated for 24 h. The cells were transfected with siRNAs or treated with CAPE, Fer-1, Compc, or TIC10 alone or in combination for 5 days. Colonies were fixed with 4% paraformaldehyde for 15 min and then stained with 0.05% Crystal Violet Staining Solution (cat. no. C0121, Beyotime Biotechnology) at room temperature for 15 min. Images of the stained colonies were captured using a Nikon camera.

### Western blot analysis

MDA-MB-231 and MDA-MB-468 cells were seeded in 6-well plates, incubated for 24 h, and treated as described in individual experiments. Samples were lysed using a cell lysis buffer for Western and IP (cat. no. P0013, Beyotime Biotechnology). Lysates were separated by 10% SDS-PAGE and transferred to PVDF membranes. Each membrane was blocked for 1.5 h at room temperature using 5% nonfat dry milk. The membranes were incubated overnight at 4°C with specific primary antibodies: cleaved Caspase-3 (cat. no. 9664, Cell signaling technology), PARP (cat. no. 9532, Cell signaling technology), Bcl-xl (cat. no. 2764, Cell signaling technology), AMPK (cat. no. 2532, Cell signaling technology), phospho-AMPK (Thr172) (cat. no. 50081, Cell signaling technology), Foxo3 (cat. no. 12829, Cell signaling technology), GAPDH (cat. no. 60004, Proteintech), GPX4 (cat. no. ER1803-15, HUABIO), Ferritin (cat. no. R1601-9, HUABIO) and Lamin B (cat. no. 12987, Proteintech). Immunoreactive bands were detected by incubating with the appropriate secondary antibodies: HRP-conjugated Affinipure Goat Anti-Mouse IgG (cat. no. SA00001-2, Proteintech) and HRP-conjugated Affinipure Goat Anti-Rabbit IgG (cat. no. SA00001-2, Proteintech). The signals were visualized using enhanced chemiluminescence reagents (Bio-Rad). Protein signals were quantified using ChemiDoc XRS+ software (Bio-Rad).

### Immunofluorescence for Foxo3 detection

MDA-MB-231 and MDA-MB-468 cells were seeded in 24-well plates, incubated for 24 h, and then treated with CAPE. The medium was removed, and the cells were washed twice with PBS. The cells were fixed with cold methanol for 10 min, blocked with 1% BSA for 30 min, incubated with anti-Foxo3 antibody (1:200) overnight at 4°C, and then incubated with phycoerythrin (PE)-labeled secondary antibody for 1 h. The nuclei were then stained with DAPI for 5 min before the images were viewed under a fluorescence microscope (400×, amplification; Nikon).

### Preparation of nuclear and cytoplasmic extracts

MDA-MB-231 and MDA-MB-468 cells were seeded in a 60 mm dish, incubated for 24 h, and treated as specified in individual experiments. Nuclear and cytoplasmic proteins were extracted from the cells by a Nuclear and Cytoplasmic Protein Extraction Kit (cat. no. P0027, Beyotime Biotechnology,). The protein concentration was determined using a BCA protein assay kit. The extracted nuclear and cytoplasmic proteins were then used for western blot analysis.

### Annexin V-FITC/PI staining for apoptosis analysis

MDA-MB-231 cells were seeded in a 60 mm dish, treated as described in individual experiments. The cells were trypsinized, collected, and washed twice with PBS. The cells were then resuspended in the binding buffer provided in the Annexin V-FITC Apoptosis Detection Kit (cat. no. C1062, Beyotime Biotechnology) to a concentration of 3 × 10^6^ cells/mL. The cells were then stained with Annexin V-FITC and PI to detect apoptosis rates according to the manufacturer’s protocol and analyzed using flow cytometry.

### TUNEL staining for apoptosis analysis

MDA-MB-231 and MDA-MB-468 cells were seeded in 24-well plates and treated as specified in the individual experiments. The cells were subjected to the terminal deoxynucleotidyl transferase-mediated FITC-dUTP nick end labeling (TUNEL) apoptosis detection assay using a one-step TUNEL Apoptosis Assay Kit (cat. no. C1086, Beyotime Biotechnology) according to the manufacturer’s instructions. TUNEL-positive cells were visualized under a fluorescence microscope at 200× amplification (Nikon).

### ROS measure

MDA-MB-231 and MDA-MB-468 cells were seeded in a 35 mm dish, and treated as described in individual experiments. Cells were labeled with DCFH-DA (cat. no. S0033S, Beyotime Biotechnology) for 30 min under 37°C and washed with PBS. The cells were then imaged under a fluorescence microscope at 100× amplification (Nikon).

### Mitochondrial membrane potential assay

MDA-MB-231 cells were seeded in a 35 mm dish, and treated as outlined in the individual experiments. Cells were then stained using a JC-1 staining kit (cat. no. C2006, Beyotime Biotechnology) according to the manufacturer’s protocol. Following this, the cells were visualized under a fluorescence microscope at 200× amplification (Nikon, Japan).

### MDA, SOD, and GSH detection

MDA-MB-231 cells were seeded in a 60 mm dish, and treated as described in individual experiments. Cells were collected, lysed, and the levels of MDA, GSH, and SOD were detected using the Lipid Peroxidation MDA Assay Kit (cat. no. S0131S, Beyotime Biotechnology), Cellular Glutathione Peroxidase Assay Kit (cat. no. S0052, Beyotime Biotechnology), and Total Superoxide Dismutase Assay kit (cat. no. S0101S, Beyotime Biotechnology), respectively, according to the manufacturer’s instructions. The absorbance was measured using an ELISA microplate reader.

### Lipid ROS assay

MDA-MB-231 and MDA-MB-468 cells were seeded in a 35 mm dish, and treated as described in individual experiments. C11-BODIPY (cat. no. L267, DOJINDO) was used for lipid ROS level detection, and the resulting images were viewed under a fluorescence microscope at 200× amplification (Nikon).

### Cell transfection

MDA-MB-231 and MDA-MB-468 cells were transfected with Negative Control (NC) and Small interfering RNA (siRNA) specifically targeting AMPK (cat. no. siB09917111034-1-5, RiboBio) and Foxo3 (at. no. siB14310162648-1-5, RiboBio) using Lipofectamine 2000 (Invitrogen).

### Animal

Female BALB/c nude mice (5 weeks, 15-18g) were purchased from the GemPharmatech Biotechnology Co., LTD. These nude mice were raised in an institutional facility under specific pathogen-free (SPF) regulations. The environmental conditions were 12 h day/night cycle, temperature 22 ± 2°C, relative humidity 55 ± 10%, standard diet, and water ad libitum.

### Ethics statement

The animal experiments were approved by the Animal Ethics Committee of Zhejiang Cancer Hospital (2022-04-044) and conformed to the principles presented in the Guidelines for the Care and Use of Laboratory Animals by the US National Institutes of Health and ARRIVE guideline. The personnel conducting animal experiments possess certification in laboratory animal practice and have undergone relevant training, encompassing knowledge of the care, management, and utilization of laboratory animals, as well as familiarity with the ethical considerations and regulatory requirements pertaining to laboratory animals.

### MDA-MB-231 tumor xenografts

MDA-MB-231 cells were resuspended in RPMI-1640 medium, and 0.1 mL of cell suspension (1×10^6^ cells) was injected subcutaneously into the right axilla of three female BALB/c nude mice. When the xenograft tumors were 1 cm^3^ in volume, the tumors were taken out and cut into 3 mm diameter pieces and transplanted subcutaneously into the right axilla of twelve female nude mice. One week later, twelve female nude mice were randomly divided into two groups, one group of mice was intraperitoneally injected with CAPE (15mg/kg, 100ul) every three days six times. In another group of mice was intraperitoneally injected with an equal volume of saline. Body weight and tumor volume were recorded at the beginning of dosing and were measured three times a week. Tumor volume was calculated as tumor volume = Length × Width2/2. In this study, no mice died before the endpoint. Mice receiving tumor transplantation were euthanized after 25 days in the event of rapid tumor growth. All animals were euthanized via cervical dislocation. Experiments were terminated early if mice exhibited severe symptoms or pain to minimize suffering. Euthanasia criteria included:1) A tumor diameter exceeding 15 mm. 2) Tumor burden surpassing 15% of body weight. 3) A body weight loss of 10%., the mice were euthanized by cervical dislocation.

### Statistical analysis

All experiments were performed at least in triplicates. Data were analyzed using the Prism 5 Program. Values are presented as the mean ± SD. Statistical significance was evaluated using a one-way analysis of variance (ANOVA) followed by Tukey’s test. The P-value of < 0.05 was regarded as statistically significant.

## Results

### CAPE inhibited cell proliferation and induced cell apoptosis

**Fig 1:**
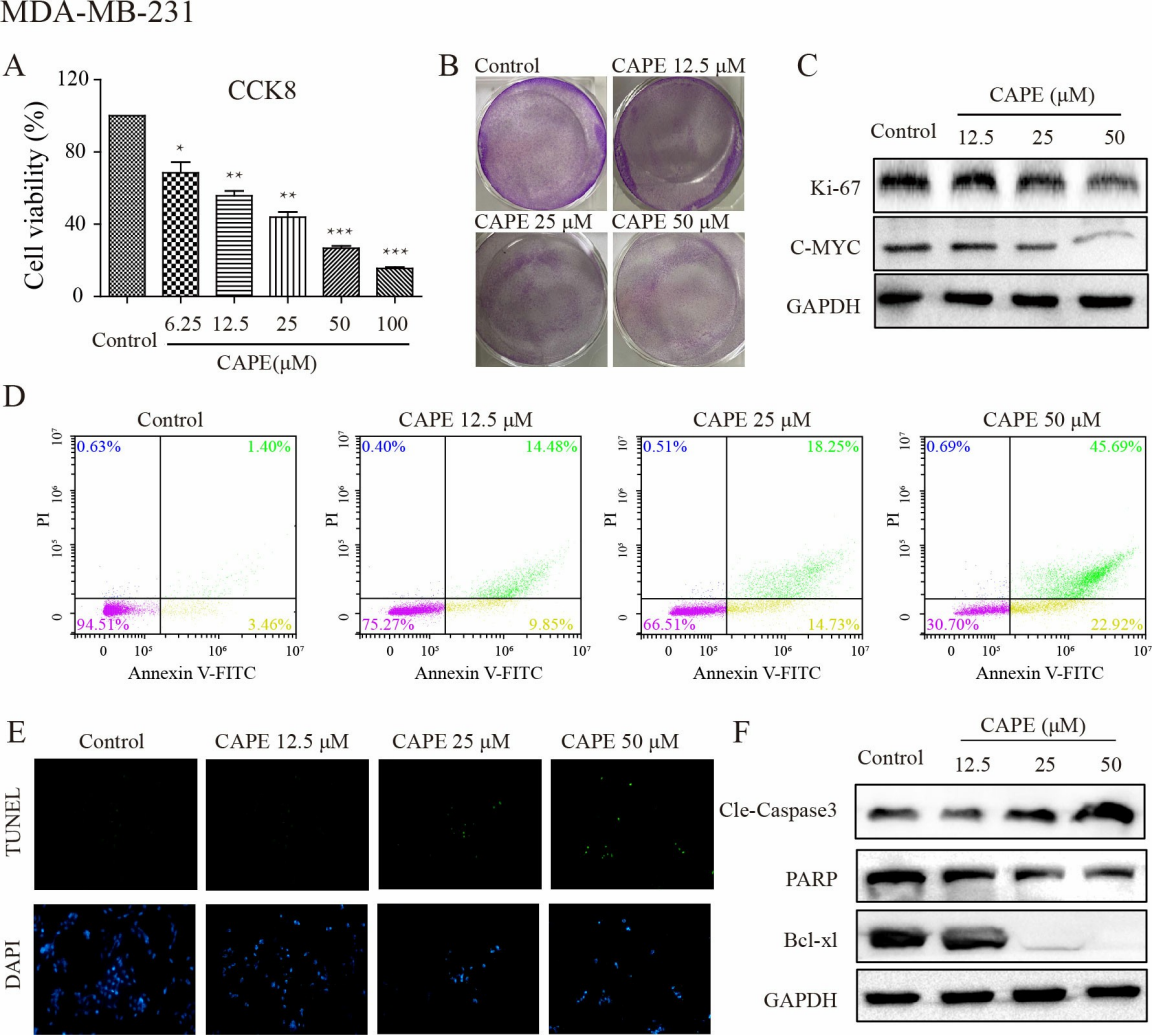
CAPE inhibited cell proliferation and induced cell apoptosis of MDA-MB-231 cells. (**A**) Cells were treated with different concentrations of CAPE (6.25–100 μM) for 72 h, and CCK8 was used for cell viability detection. (**B**) Cell were treated with CAPE (12.5, 25, 50 μM) for 5 days to investigate cell colony formation. (**C**) The inhibition of cell proliferation was measured by western blot for the level of Ki-67 and C-MYC using CAPE (12.5, 25, 50 μM) treatment for 24 h. (**D**) Cells were treated with CAPE (12.5, 25, 50 μM) for 48 h, and the cell apoptosis was detected by TUNEL/DAPI dual staining. (**E**) Cells were treated with CAPE (12.5, 25, 50 μM) for 24 h, and western blot was used to detect the expression of Cle-Caspase3, PARP, and Bcl-xl. (**F**) Cells were treated with CAPE (12.5, 25, 50 μM) for 48 h, and apoptosis was determined by Annexin V-FITC/PI dual staining. Values represent the mean ± SD from three independent experiments; *p <0.05, **p<0.01, ***p<0.001: CAPE groups compared with the control group.

To investigate the effect of CAPE on cell proliferation. The MDA-MB-231 and MDA-MB-468 cells were treated with different concentrations of CAPE, and stained by CCK8. As shown in Fig 1A and S1A Fig, cell viability decreased as the concentration of CAPE increased, from 70.2% for a dose of 6.25 μM to 62.8% for 12.5 μM, to 45.6% at 25 μM, 27.4% at 50 μM, and finally to 17.4% at 100 μM. We chose three concentrations of CAPE (12.5, 25, and 50 μM) for subsequent experiments. Cell colonies were detected, as shown in Fig 1B and S1B Fig, the colonies in the control group were larger and numerous while in the CAPE-treated groups there were fewer and smaller colonies. Ki-67 and c-MYC are two classic indicators associated with cell proliferation. As shown in Fig 1C, the protein levels of Ki-67 and c-MYC detected by western blot were decreased in CAPE-treatment groups. Additionally, to determine the effect of CAPE on cell apoptosis, we incubated cells with CAPE at 12.5, 25, and 50 μM and analyzed cell apoptosis. The TUNEL test in Fig 1D and S1C Fig showed CAPE treatment induced cell apoptosis. Western blot assay was used to detect apoptotic proteins. As shown in Fig 1E and S1D Fig, the level of cleaved caspase 3 (Cle-caspase 3), which is the most important terminal shear enzyme in apoptosis, was significantly increased, while the level of PARP, a well-known substrate for caspase-3, was remarkably decreased as well as Bcl-xl after receiving CAPE. Bcl-xl is an anti-apoptotic protein in the Bcl-2 family that regulates cell apoptosis. Previously published studies showed that CAPE could decrease Bcl-xl expression levels [20]. In addition, we observed that CAPE treatment induced cell death mainly through late apoptosis, as analyzed by Annexin V-FITC/PI staining using flow cytometry (Fig 1F). The treatment significantly decreased the number of live cells. Our results showed that CAPE exhibited a dose-dependent ability to inhibit cell proliferation and induce cell apoptosis.

### CAPE induced cell ferroptosis

**Fig 2:**
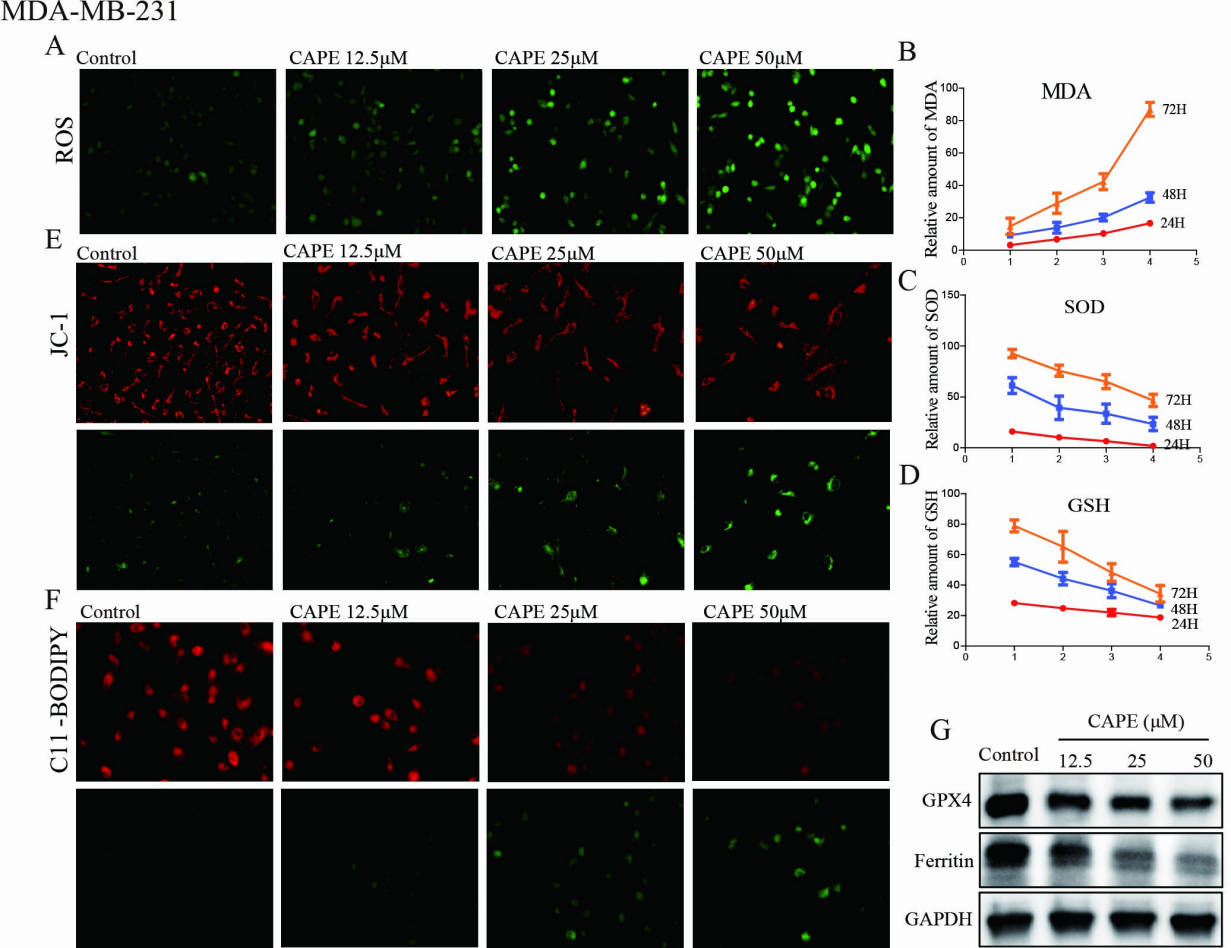
CAPE induced cell ferroptosis of MDA-MB-231 cells. (**A**) Cells were treated with CAPE (12.5, 25, 50 μM) for 24 h, and DCFH-DA was used for ROS detection. (**B-D**) Cells were treated with CAPE (12.5, 25, 50 μM) for 24 h to investigate MDA, SOD and GSH levels. (**E**) Cells were treated with CAPE (12.5, 25, 50 μM) for 24 h, and JC-1 staining was used to detect the mitochondrial membrane potential. (**F**) Cells were treated with CAPE (12.5, 25, 50 μM) for 24 h, and the lipid peroxidation was determined by C11-BODIPY staining. (**G**) Cells were treated with CAPE (12.5, 25, 50 μM) for 24 h, and western blot was used to detect the expression of GPX4 and Ferritin. Values represent the mean ± SD from three independent experiments.

Ferroptosis, a recently discovered type of cell death, is characterized by excessive intracellular lipid oxidation, elevated levels of reactive oxygen species (ROS), and diminished mitochondrial activity. The MDA-MB-231 and MDA-MB-468 cells were exposed to CAPE at concentrations of 12.5, 25, and 50 μM to investigate whether it induced ferroptosis. Initially, we measured the levels of ROS, malondialdehyde (MDA), superoxide dismutase (SOD), and glutathione (GSH). As illustrated in Fig 2A–2D and S2A Fig following CAPE treatment, there was a steady increase in ROS fluorescence and MDA levels compared to the control group, while SOD activity and GSH consumption decreased. Furthermore, the JC-1 probe was utilized to evaluate the potential and function of the mitochondrial membrane. Fig 2E illustrated that greater green fluorescence in response to 12.5, 25, and 50 μM CAPE indicated a lower mitochondrial membrane potential, indicating mitochondrial dysfunction. Subsequently, the lipid peroxidation of cells was examined by C11-BODIPY labeling, and it was found that the lipid ROS in Fig 2E and S2B Fig was greatly increased by CAPE treatment. Ferritin is a cellular iron storage protein, and diminished expression of ferritin leads to an increase in intracellular iron concentration, thereby promoting ferroptosis. GPX4 is capable of degrading small molecule peroxides as well as certain lipid peroxides, thereby inhibiting lipid peroxidation. It has been observed that cells with down-regulated expression of GPX4 exhibit increased sensitivity to ferroptosis. Compared to the control group, the Ferritin and GPX4 levels were found to be lower in groups treated with CAPE as depicted in Fig 2G and S2C Fig. Our results indicated that CAPE exhibited a dose-dependent ability to induce cell ferroptosis.

### Fer-1 counteracted the antitumor efficacy of CAPE

**Fig 3:**
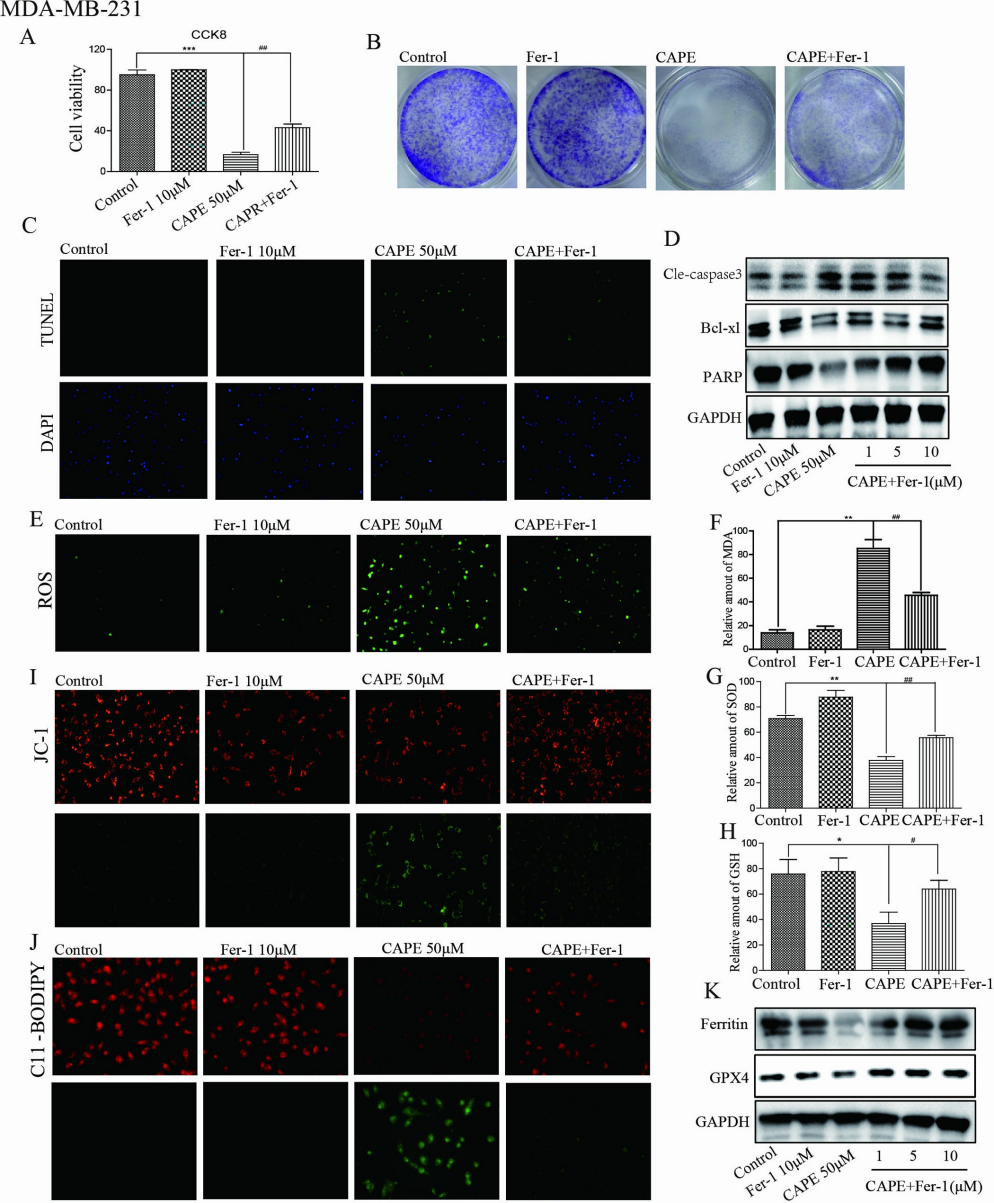
Fer-1 counteracted the antitumor efficacy of CAPE. (**A-D**) Cells were treated singly or in combination with Fer-1 (10 μM) and CAPE (50 μM), and cell viability was determined by CCK8 at 72 h. colony formation assay was performed after 5 days. TUNEL staining and western blot were conducted for apoptotic cells and apoptosis-related proteins at 24 h. (**E-I**) Cells were treated singly or in combination with Fer-1 (10 μM) and CAPE (50 μM) for 24 h, DCFH-DA was used for ROS detection. MDA, SOD and GSH levels were detected using ELISA kits. JC-1 staining was used to detect the mitochondrial membrane potential. (**J-K**) Cells were treated with singly or in combination with Fer-1 (10 μM) and CAPE (50 μM) for 24 h, and the lipid peroxidation was determined by C11-BODIPY staining. The expression of GPX4 and Ferritin were detected using western blot.Values represent the mean ± SD from three independent experiments. *p<0.05, **p<0.01, ***p<0.001: CAPE groups compared with the control group; ^#^p<0.01, ^##^p<0.01: CAPE+Fer-1 groups compared with the CAPE group.

To ascertain whether ferroptosis contributes to the anticancer effects of CAPE, we utilized the ferroptosis-specific inhibitor Fer-1. Treatment with Fer-1 (10 μM) mitigated CAPE’s inhibition of proliferation and induction of apoptosis in MDA-MB-231 and MDA-MB-468 cells. The results from CCK8 and cell colony assays (Fig 3A, 3B and S2D, S2E Fig) demonstrated that the cell viability was higher and the number of colonies was larger in the CAPE + Fer-1 group compared to the CAPE group. Additionally, TUNEL staining and western blot assays shown in Fig 3C, 3D and S2F, S2G Fig revealed that Fer-1 treatment reduced both the number of apoptotic cells and the level of apoptotic protein Cle-caspase 3 induced by CAPE; however, it also resulted in increased levels of PARP and Bcl-xl in the CAPE+Fer-1 group compared to the CAPE group. Subsequently, we investigated the impact of Fer-1 on ferroptosis. As illustrated in Fig 3E–3H and S2H Fig, Fer-1 (10 μM) mitigated the downregulation effects on SOD and GSH as well as the upregulation effects on ROS and MDA induced by CAPE. Regarding mitochondrial dysfunction, the JC-1 staining results were shown in Fig 3I. Lipid peroxidation induced by CAPE was reduced by Fer-1 treatment, as seen in Fig 3J and S2I Fig. Furthermore, Fer-1 mitigated the downregulation of GXP4 and Ferritin that induced by CAPE, as shown in Fig 3K and S2J Fig. Our results indicated that ferroptosis serves as an intermediary regulatory pathway for CAPE’s anti-tumor effect.

### CAPE activated AMPK and Foxo3 signals

**Fig 4:**
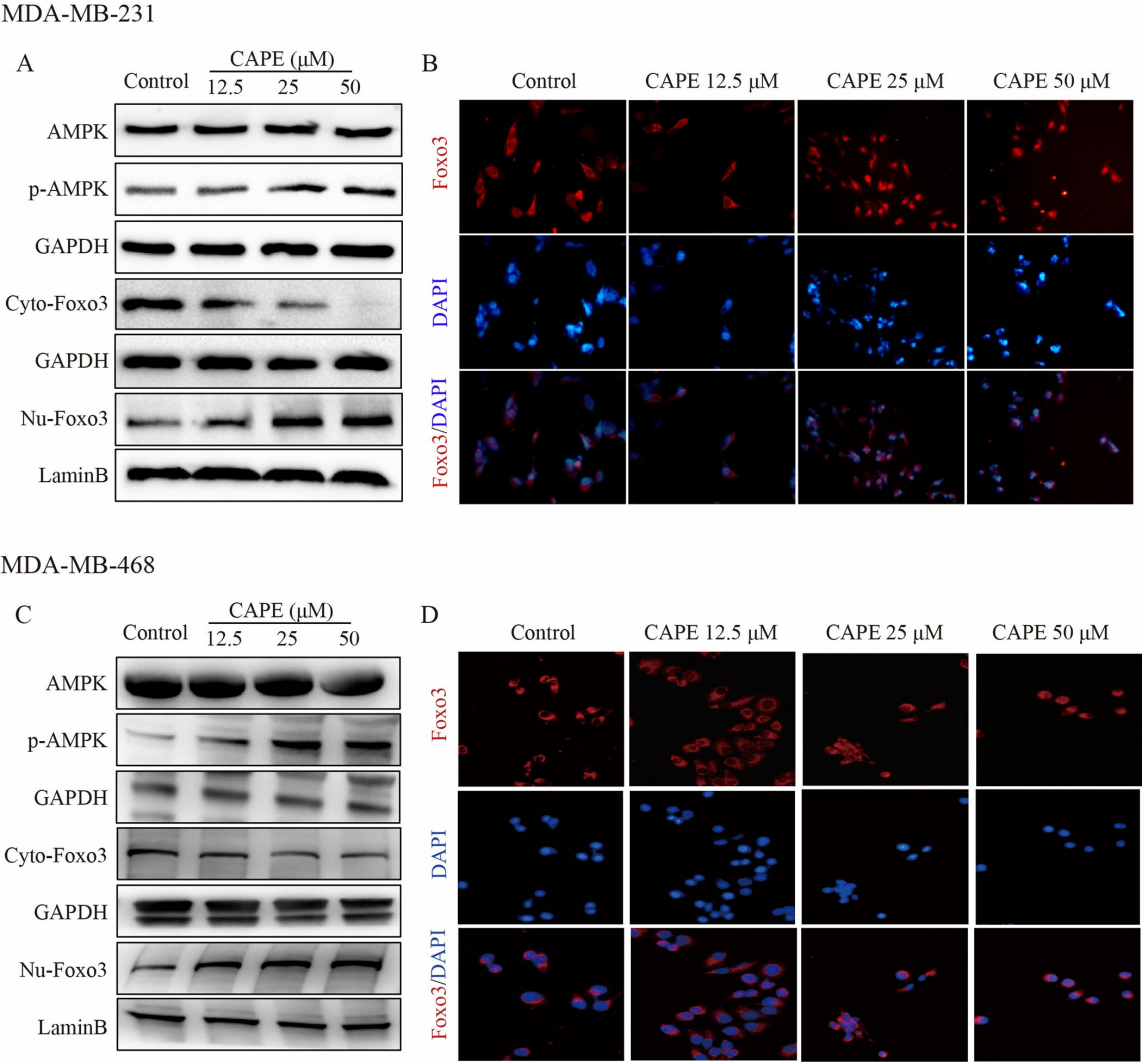
CAPE activated the AMPK and Foxo3 signals. (**A, C**) MDA-MB-231 and MDA-MB-468 cells were treated with CAPE (12.5, 25, 50 μM) for 12 h, and the protein levels of p-AMPK, GAPDH, Foxo3 (respectively in the cytoplasm and nucleus), and LaminB were analyzed by western blot. (**B, D**) MDA-MB-231 and MDA-MB-468 cells were treated with CAPE (12.5, 25, 50 μM) for 12 h, and the nuclear translocation of Foxo3 was determined by immunofluorescence staining. Values represent the mean ± SD from three independent experiments.

AMPK is closely correlated to the tumor-suppressive functions of P53 and LKB1; consequently, modulating the activity of cell survival signaling pathways such as Akt and mTOR leads to inhibition of cell growth. AMPK can mediate cell apoptosis by regulating oxidative stress and autophagy [27]. As shown in Fig 4A and 4C, AMPK was activated by CAPE treatment in MDA-MB-231 and MDA-MB-468 cells. The phosphorylation level of AMPK increased in a dose-dependent manner upon incubation with CAPE at concentrations of 12.5, 25, and 50 μM. Foxo3 functions downstream of the PI3K-Akt signaling cascade and is crucial for cell survival, cell proliferation, senescence, cell cycle control, and DNA-damage repair. Nuclear translocation of Foxo3 is an important manifestation of its signal activation. As shown, the protein level of Foxo3 in the cytoplasm decreased while the level in the nucleus increased after treatment with CAPE, indicating that Foxo3 was migrating from the cytoplasm to the nucleus. We obtained consistent results from the immunofluorescence experiment depicted in Fig 4B and 4D. Our findings implied that CAPE may activate AMPK and Foxo3 signals.

### Repression of AMPK diminished the anticancer efficacy of CAPE

**Fig 5:**
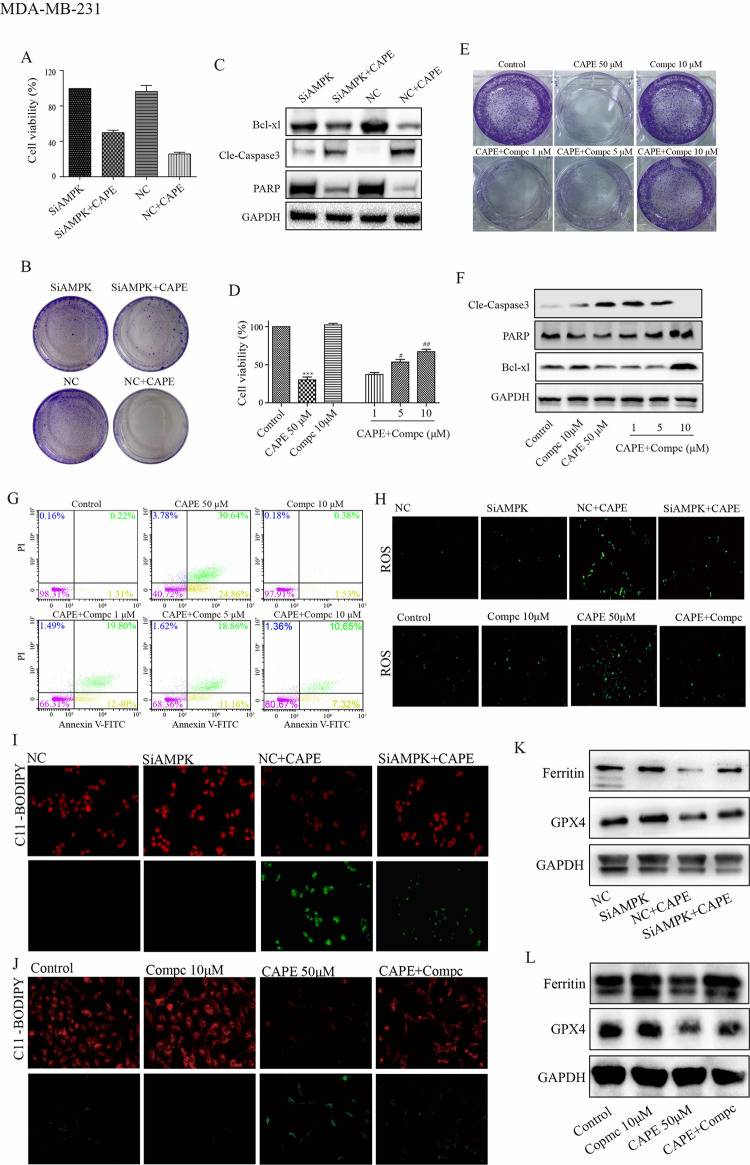
Inhibition of AMPK signal reversed the antitumor effects of CAPE in MDA-MB-231 cells. (**A-C**) Cells were transfected with siRNA, and the cell viability was determined by CCK8 at 72 h, the colony was investigated by colony formation assay at 5 days and the expression of Cle-caspase3, PARP, and Bcl-xl were detected by western blot at 24 h. (**D-G**) Cells were treated singly or in combination with Compc (1, 5, 10 μM) and CAPE (50 μM) for 72 h. Cell viability was determined by CCK8. The colony was investigated by colony formation assay at 5 days. The expression of Cle-caspase3, PARP, and Bcl-xl were detected by western blot at 24 h. Apoptosis was determined by Annexin V-FITC/PI dual staining at 48 h. (**H-L**) Cells were transfected with siRNA or treated in combination with Compc (10 μM) for 24 h, DCFH-DA was used for ROS detection. The lipid peroxidation was determined by C11-BODIPY staining. The expression of GPX4 and Ferritin were detected using western blot. Values represent the mean ± SD from three independent experiments. ***p<0.001: CAPE compared with control, ^#^p <0.05, ^##^p<0.01: combinatorial Compc+CAPE groups compared with the single CAPE group.

To confirm the mediating role of AMPK in the anti-tumor effect of CAPE, we transfected cells with siRNA targeting AMPK or treated cells with Compc, an inhibitor of AMPK that functions by preventing its phosphorylation in MDA-MB-231 and MDA-MB-468 cells (S3A, S3C and S3E Fig). As shown by CCK8 and cell colony assays in Fig 5A, 5B and S4A, S4B Fig, the NC group treated with CAPE exhibited a significant inhibition of cell proliferation compared with NC or SiAMPK groups alone; however, when SiAMPK cells were treated with CAPE, their inhibitory effect on cell proliferation was diminished. We also detected apoptosis related proteins. The results indicated that CAPE significantly increased Cle-caspase3 levels but decreased PARP and Bcl-xl levels in the NC-transfected group; however, these effects were attenuated in the SiAMPK-transfected group (Fig 5C and S4C, S4D Fig). Also, results generated by Compc showed that CAPE alone significantly inhibited cell proliferation compared with control and Compc alone; however, when cells were incubated with CAPE and Compc together, the inhibition effect on proliferation of CAPE was weakened and related inversely to Compc concentration (Fig 5D and 5E). Additionally, we employed Annexin V-FITC/PI staining and western blot to investigate the effect of Compc on CAPE-induced cell apoptosis. As shown in Fig 5F and 5G, CAPE had a significant effect on apoptosis cells and apoptosis related proteins. Similar to its effects on cell proliferation, co-incubating of Compc with CAPE reduced the percentage of apoptotic cells and increased the levels of PARP, and Bcl-xl. Additionally, it led to a decrease in Cle-caspase3 levels compared to CAPE alone. Subsequently, we investigate how the suppression of AMPK influences ferroptosis caused by CAPE. The results from the ROS, MDA, SOD, and GSH experiments demonstrated that AMPK inhibition achieved by both siRNA and Compc reduced cell oxidation induced by CAPE (Fig 5H, S4E and S5A–S5C Figs). Subsequently, we employed JC-1 staining to evaluate mitochondrial function. The increased membrane potential observed in the CAPE+Compc compared to the CAPE group was indicated by a reduction in green fluorescence intensity (S5D Fig). Furthermore, both siRNA and Compc mitigated the effects of CAPE on lipid peroxidation revealed by C11-BODIPY staining (Fig 5I and 5J, S4F Fig). Furthermore, as Fig 5K, 5L and S4G Fig illustrated, Ferritin and GPX4 levels were elevated in the CAPE+AMPK-inhibition groups compared to the CAPE groups. Our results indicate that the inhibition of AMPK can reverse the anti-tumor effect of CAPE, suggesting that AMPK plays a crucial role as an anti-tumor mechanism for CAPE.

### Repression of Foxo3 diminishes the antitumor efficacy of CAPE

**Fig 6:**
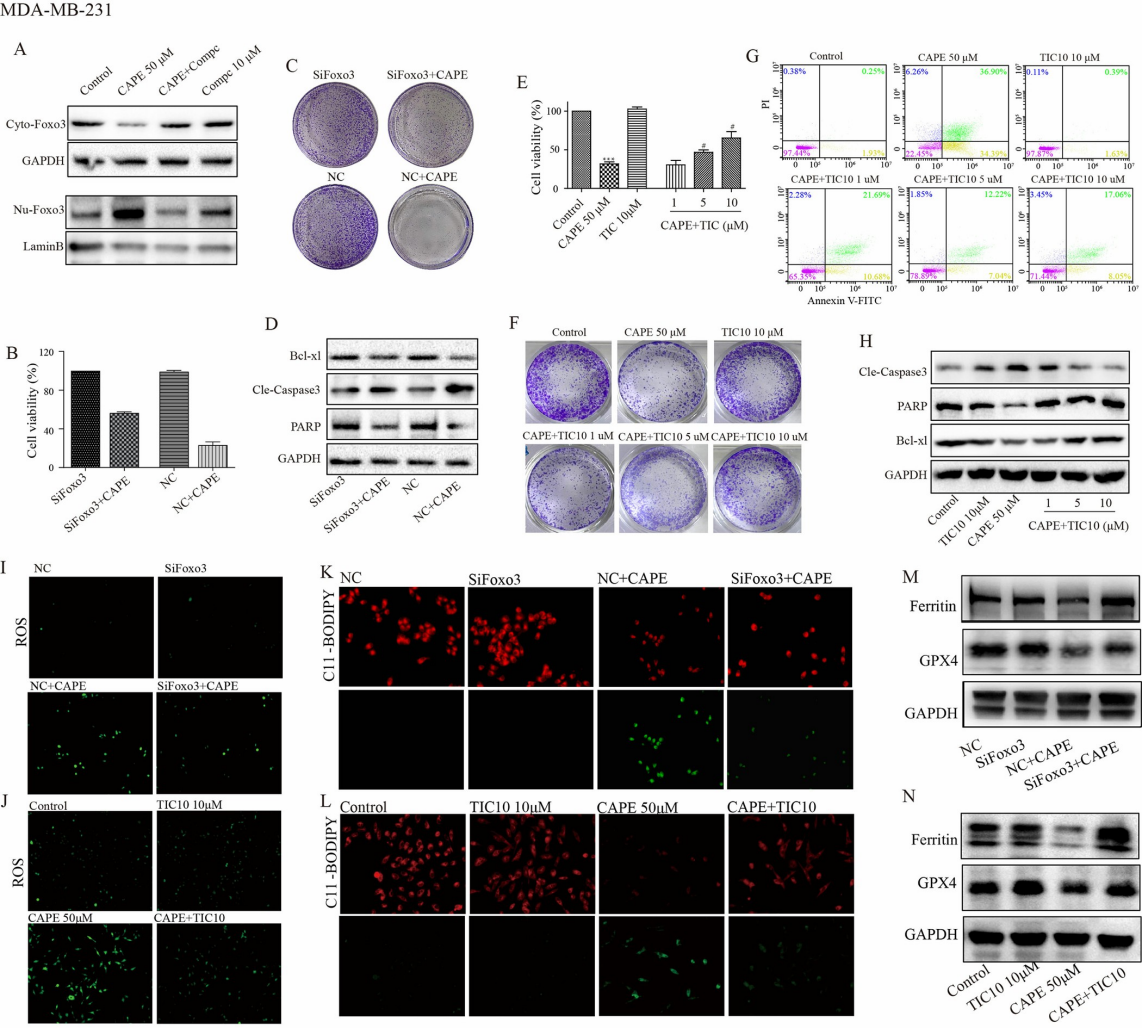
Inhibition of Foxo3 signal reversed the antitumor effects of CAPE in MDA-MB-231 cells. (**A**) Cells were treated singly or in combination with Compc (10 μM) and CAPE (50 μM) for 12 h, and western blot was used for cytoplasm and nuclear Foxo3 detection. (**B-D**) Cells were transfected with siRNA, and CCK8 was used for the cell viability detection at 72 h, the colony was investigated by colony formation assay at 5 days and the expression of Cle-caspase3, PARP, and Bcl-xl were detected by western blot at 24 h. (**E-H**) Cells were treated singly or in combination with TIC10 (1, 5, 10 μM) and CAPE (50 μM) for 72 h. The cell viability was determined by CCK8. The colony was investigated by colony formation assay at 5 days. Apoptosis was determined by Annexin V-FITC/PI dual staining at 48 h. The expression of Cle-caspase3, PARP, and Bcl-xl were detected by western blot at 24 h. (**I-N**) Cells were transfected with siRNA or treated in combination with TIC10 (10 μM) for 24 h, DCFH-DA was used for ROS detection. The lipid peroxidation was determined by C11-BODIPY staining. The expression of GPX4 and Ferritin were detected using western blot. Values represent the mean ± SD from three independent experiments; ***p<0.001: CAPE compared with control, ^#^p <0.05: combinatorial TIC10+CAPE groups compared with the single CAPE group.

Foxo3 is a downstream molecule of AMPK. As shown in Fig 6A, inhibition of AMPK by Compc (10 μM) completely abolished the CAPE-induced nuclear transfer of Foxo3, indicating that Foxo3 may serve as a downstream mechanism of CAPE. CCK8 and cell colony assays shown that CAPE significantly inhibited cell proliferation in the NC-transfected group; however, the effect was diminished in SiFoxo3-transfected group (Figs 6B, 6C, S4H and S4I). Subsequently, we evaluated cell apoptosis through Cle-caspase3, PARP and Bcl-xl analysis. As shown in Fig 6D and S4J, S4K Fig, treatment with CAPE resulted in increased levels of Cle-caspase3 and decreased levels of PARP and Bcl-xl in the NC-transfected; conversely, these effects were attenuated in the SiFoxo3-transfected group. TIC10 inhibits Foxo3 by obstructing its nuclear transfer (S3D Fig). CCK8 and cell colony assays indicated that TIC10 could resist the effect of CAPE on cell proliferation in a dose-dependent manner. As depicted in Fig 6E, 6F, cells incubation with both TIC10 and CAPE exhibited enhanced viability along with larger and more abundant colonies compared to those treated with CAPE alone. Furthermore, TIC10 reversed apoptotic induction caused by CAPE. As presented in Fig 6G and 6H, cells co-treated with TIC10 and CAPE displayed a reduction in apoptotic cells alongside decreased expression of pro-apoptotic protein cle-caspase3 while showing an increase in both the pro-apoptotic substrate PARP expression as well as the anti-apoptotic protein Bcl-xl compared to treatment with CAPE alone. Subsequently, we investigated the impact of Foxo3 suppression on ferroptosis induced by CAPE. The results from experiments measuring ROS, MDA, SOD, and GSH demonstrated that Foxo3 inhibition achieved through both siRNA and TIC10 effectively reduced cell oxidation triggered by CAPE (Fig 6I, 6J; S4L; S5E–S5G Figs). We then employed JC-1 staining to assess mitochondrial function. The observed increase in membrane potential in the CAPE+TIC10 group compared to the CAPE group was indicated by a reduction in green fluorescence intensity (S5H Fig). Furthermore, both siRNA and TIC10 alleviated the effects of CAPE on lipid peroxidation as revealed by C11-BODIPY staining (Fig 6K and 6L; S4M Fig). Additionally, as illustrated in Fig 6M, 6N and S4N Fig, levels of Ferritin and GPX4 were significantly elevated in the CAPE+AMPK-inhibition groups relative to those in the CAPE groups. Our results suggest that Foxo3 represents another critical anti-tumor mechanism associated with CAPE while confirming that AMPK functions upstream of Foxo3.

### CAPE inhibited tumor growth in nude mice

**Fig 7:**
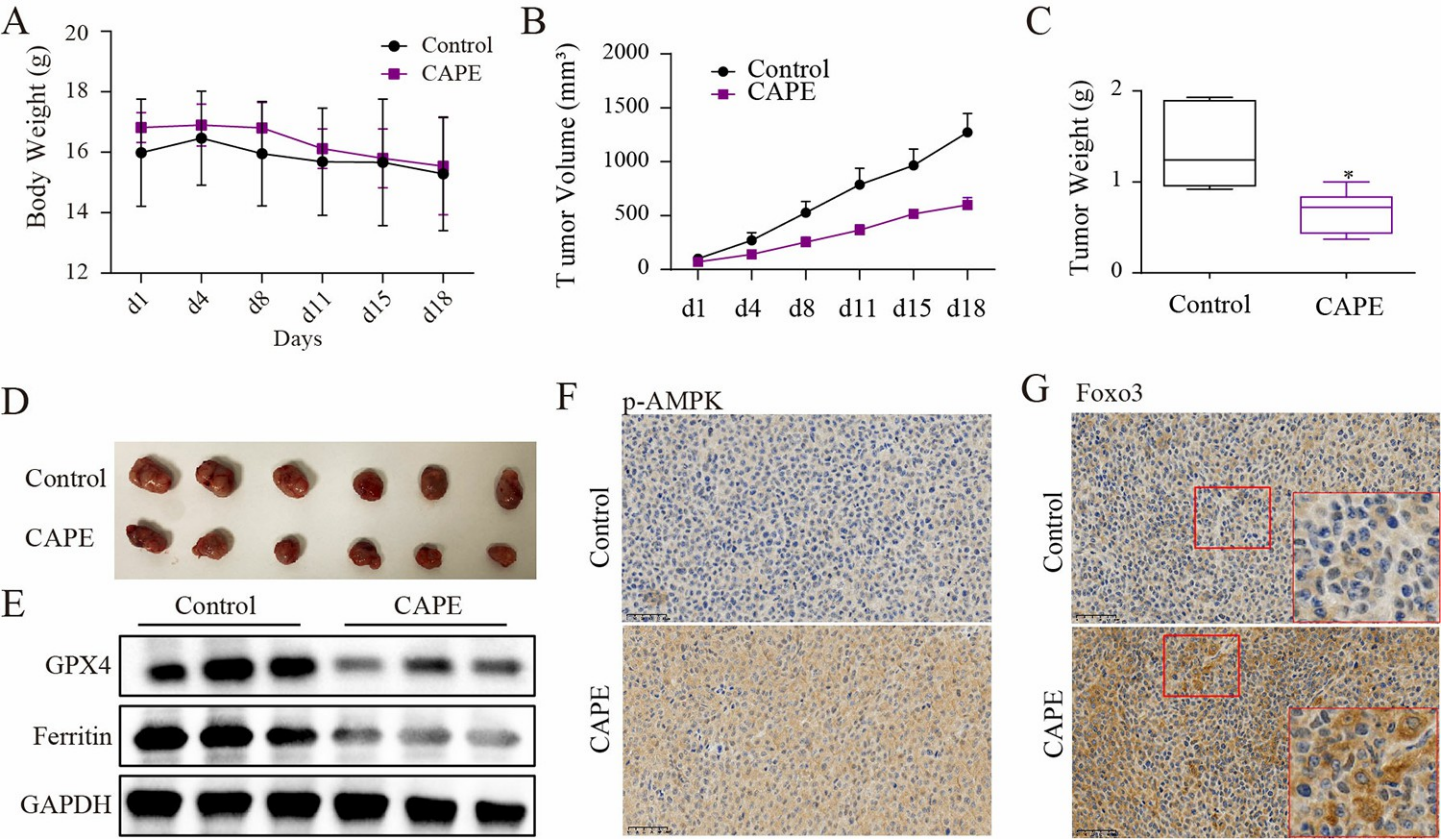
CAPE administration inhibited tumor growth of nude mice. (**A**) Mice were treated with CAPE (15mg/kg) via tail vein injection, and the body weight was recorded of the Control-group and the CAPE-group. (**B**) Tumor sizes were presented using photographs of the Control-group and the CAPE-group. (**C**) Tumor volumes were recorded for both the Control-group and CAPE-group. (**D**) Tumor weights were measured for both the Control-group and CAPE-group. (**E**) Expression of GPX4 and Ferritin were detected of Ctrol-group and CAPE-group. (**F-G**) The levels of p-AMPK and nuclear Foxo3 were tested. Values represent the mean ± SD from the mice; *p<0.05: CAPE compared with control.

To confirm the anti-tumor effect of CAPE in vivo, *nude mice* were transplanted with tumor tissue to establish a subcutaneous breast cancer model. Animals in the two groups showed similar body weights during the period of drug administration (Fig 7A). However, there were significant differences in tumor size between the two groups (Fig 7B–7D). The xenografts treated with CAPE showed a smaller tumor volume and a lower growth rate. And at the end of the experiment, xenografts from the CAPE group had a lighter tumor weight. Furthermore, the administration of CAPE resulted in ferroptosis within tumors, as evidenced by the down-regulation of GPX4 and Ferritin expression (Fig 7E). The immunohistochemical results demonstrated that the administration of CAPE could enhance the phosphorylation of AMPK in tumor tissues and facilitate the nuclear translocation of Foxo3 (Fig 7F and 7G). Our results suggest that CAPE has anti-breast cancer effects and may be a potential drug.

## Discussion

CAPE is a phenolic ingredient in propolis, a resinous substance produced by bees and a concentrated source of polyphenols. This compound has garnered significant interest as an effective anticancer agent due to its demonstrated ability to inhibit the progression of various tumors, including colorectal, prostate, and liver [28]. As the primary component of propolis. In the present study, we found that CAPE significantly inhibited proliferation and promoted ferroptosis of TNBC cells in a dose-dependent manner. Furthermore, it displayed remarkable inhibitory effects on tumors in TNBC xenografts implanted in nude mice, suggesting that CAPE might be a potentially safe and effective option for treating TNBC.

AMPK inactivation has been implicated in numerous malignancies, disrupting multiple signalling pathways and thereby contributing to tumor progression. Conversely, AMPK activation has a negative effect on cell growth because of the inhibition of AKT target [29]. Activation of AMPK leads to decreased expression levels of EGFR, cyclin D1, and cyclin E while also inhibiting the phosphorylation of mitogen-activated protein kinase (MAPK), Src, signal transducers and transcriptional activator 3 (STAT3), all of which are associated with cell survival [30–32]. Additionally, AMPK activation enhances autophagy and ROS through activating mTOR and AKT signaling pathways, ultimately leading to ferroptosis [33]. Moreover, AMPK activation is critical in alleviating metabolic and energetic stresses associated with tumor progression. The loss of AMPK signal transduction can enhance oncogenic drivers such as LKB1, TSC2, and P53, thereby promoting cell growth, proliferation, and the reprogramming of cancer cell metabolis [34, 35]. These studies suggested that AMPK could represent a viable target for CAPE’s action. We wondered whether the inhibition of AMPK could obstruct CAPE’s regulatory influence on cell proliferation and ferroptosis. We observed an increase in the phosphorylation level of AMPK induced by CAPE, indicating that AMPK may as a target for CAPE in TNBC. Furthermore, the inhibition of AMPK markedly influenced the inhibition of proliferation and induction of ferroptosis on TNBC cells, indicating the vital role of AMPK in the anti-tumor effect of CAPE.

The transcription factor Foxo3, a key determinant of cancer cell homeostasis, plays a dual role at the intersection of survival and apoptosis in response to metabolic stress and cancer chemotherapies [36, 37]. The nuclear localization and transcriptional activation of Foxo3 can be enabled by AMPK [38]. However, it remains unclear whether CAPE influences Foxo3. We isolated both nuclear and cytosolic fractions from TNBC cells treated with varying concentrations of CAPE and examined changes in the localization of Foxo3 proteins. Our findings indicate that CAPE affects the nucleocytoplasmic localization of Foxo3 by promoting its transfer into the nucleus.

The altered nucleocytoplasmic distribution of Foxo3 suggests variations in its binding efficiency to promoter regions of the target genes. Nevertheless, this observation does not conclusively establish that Foxo3 is involved in the mechanism underlying CAPE’s antitumor activity. To further investigate this relationship, we employed siRNA or TIC10, targeting Foxo3, to assess its impact on the anti-tumor effect of CAPE. Our results revealed that the effect of CAPE on the inhibition of cell proliferation and induction of cell ferroptosis was partially blocked in the presence of TIC10 or transfected with siRNA of Foxo3. Moreover, we found that AMPK blockage inhibited the nuclear translocation induced by CAPE. These data provide direct evidence that CAPE promotes the nuclear translocation of Foxo3 through AMPK activation, and Foxo3 serves as a regulatory molecule for ferroptosis and mediates the induction effect of CAPE on TNBC cells.

## Conclusion

Our study demonstrates that CAPE effectively inhibits cell proliferation and induces cell ferroptosis. CAPE exhibits minimal toxicity towards human cells with better anti-tumor effect, indicating CAPE may emerge as an effective candidate drug for clinical chemotherapy combinations. Additionally, CAPE exhibits anti-inflammatory detoxification properties along with hepatoprotective effects; it can mitigate chemotherapy toxicity while enhancing patients’ tolerance to both cycle duration and dosage, ultimately improving therapeutic outcomes. Moreover, our study elucidates that the mechanism underlying CAPE’s action involves the activation of AMPK alongside nuclear translocation of Foxo3. However, due to its low solubility and bioavailability, CAPE faces limitations regarding its application in treatment and prevention strategies. Future investigations may focus on developing formulations such as liposomal drug carriers or nano-encapsulation techniques.

## Supporting information

S1 Fig CAPE inhibited cell proliferation and induced cell apoptosis of MDA-MB-468 cells. (**A**) MDA-MB-468 cells were treated with different concentrations of CAPE (6.25–100 μM) for 72 h, and CCK8 was used for cell viability detection. (**B**) MDA-MB-468 cell were treated with CAPE (12.5, 25, 50 μM) for 5 days to investigate cell colony formation. (**C**) MDA-MB-468 cells were treated with CAPE (12.5, 25, 50 μM) for 24 h, and western blot was used to detect the expression of Cle-Caspase3, PARP, and Bcl-xl. (**D**) MDA-MB-468 cells were treated with CAPE (12.5, 25, 50 μM) for 48 h, and the cell apoptosis was detected by TUNEL/DAPI dual staining. Values represent the mean ± SD from three independent experiments; *p <0.05, ***p<0.001: CAPE groups compared with the control group.
(TIF)

S2 Fig CAPE-induced ferroptosis of MDA-MB-468 cells, while Fer-1 mitigated this effect. (**A**) Cells were treated with CAPE (12.5, 25, 50 μM) for 24 h, and DCFH-DA was used for ROS detection. (**B**) Cells were treated with CAPE (12.5, 25, 50 μM) for 24 h, and the lipid peroxidation was determined by C11-BODIPY staining. (**C**) Cells were treated with CAPE (12.5, 25, 50 μM) for 24 h, and western blot was used to detect the expression of GPX4 and Ferritin. (**D-G**) Cells were treated singly or in combination with Fer-1 (10 μM) and CAPE (50 μM), and cell viability was determined by CCK8 at 72 h. colony formation assay was performed after 5 days. TUNEL staining and western blot were conducted for apoptotic cells and apoptosis-related proteins at 48 h and 24 h. (**H-J**) Cells were treated with singly or in combination with Fer-1 (10 μM) and CAPE (50 μM) for 24 h, and the lipid peroxidation was determined by ROS and C11-BODIPY staining, along with GPX4 and Ferritin expression by western blot. Values represent the mean ± SD from three independent experiments. ***p<0.001: CAPE groups compared with the control group; ^###^p<0.001: CAPE+Fer-1 groups compared with the CAPE group.
(TIF)

S3 Fig Inhibiting efficiency of siRNAs or inhibitors targeting AMPK or Foxo3. (**A-B**) MDA-MB-231 cells were transfected with small interfering RNA targeting AMPK or Foxo3, and western blot was used to detect transfection efficiency. (**C**) MDA-MB-231 cells were treated singly or in combination with Compc (10 μM) and CAPE (50 μM) for 12 h, and the protein levels of p-AMPK and GAPDH were analyzed by western blot. (**D**) MDA-MB-231 cells were treated singly or in combination with TIC10 (10 μM) and CAPE (50 μM) for 12 h. The protein level of Foxo3 in cytoplasm and nuclear were analyzed by western blot. (**E-F**) MDA-MB-468 cells were transfected with small interfering RNA targeting AMPK or Foxo3, and western blot was used to detect transfection efficiency. Values represent the mean ± SD from three independent experiments.
(TIF)

S4 Fig Inhibiting AMPK and Foxo3 signals mitigated CAPE’s antitumor effects in MDA-MB-468 cells. (**A-D**) Cells were transfected with siRNA targeting AMPK. The CCK8 was used for the cell viability detection at 72 h. The colony was investigated by colony formation assay after 5 days. The cell apoptosis was detected by TUNEL/DAPI dual staining at 48 h. The expression of Cle-caspase3, PARP, and Bcl-xl were detected by western blot at 24 h. (**E-G**) Cells were transfected with siRNAs targeting AMPK for 24 h, DCFH-DA was used for ROS detection, C11-BODIPY staining was performed for lipid peroxidation detection and western blot was used for GPX4 and Ferritin levels test. (**H-K**) Cells were transfected with siRNA targeting Foxo3. The CCK8 was used for the cell viability detection at 72 h. The colony was investigated by colony formation assay after 5 days. The cell apoptosis was detected by TUNEL/DAPI dual staining at 48 h. The expression of Cle-caspase3, PARP, and Bcl-xl were detected by western blot at 24 h. (**L-N**) Cells were transfected with siRNAs targeting Foxo3 for 24 h, DCFH-DA was used for ROS detection, C11-BODIPY staining was performed for lipid peroxidation detection and western blot was used for GPX4 and Ferritin levels test. Values represent the mean ± SD from three independent experiments. ***p<0.001: the SiAMPK or SiFoxo3 groups compared with the NC+CAPE groups; ^##^p<0.01: the SiAMPK+CAPE or SiFoxo3+CAPE groups compared with the NC+CAPE groups.
(TIF)

S5 Fig Both Compc and TIC10 mitigated CAPE-induced cell oxidation in MDA-MB-23 cells. (**A-C**) Cells were treated singly or in combination with Compc (10 μM) and CAPE (50 μM) for 24 h to investigate ROS, MDA, SOD and GSH levels. (**D**) Cells were treated singly or in combination with Compc (10 μM) and CAPE (50 μM) for 24 h, and JC-1 staining was used to detect the mitochondrial membrane potential. (**E-G**) Cells were treated singly or in combination with TIC10 (10 μM) and CAPE (50 μM) for 24 h to investigate ROS, MDA, SOD and GSH levels. (**H**) Cells were treated singly or in combination with TIC10 (10 μM) and CAPE (50 μM) for 24 h, and JC-1 staining was used to detect the mitochondrial membrane potential. Values represent the mean ± SD from three independent experiments. *p<0.05, **p<0.01***p<0.001: CAPE compared with control, ^#^p <0.05, ^##^p<0.01: combinatorial Compc+CAPE or TIC10+CAPE groups compared with the CAPE group.
(TIF)

S1 Raw images Raw western blot scans. (PDF)
