# Supplementary figures and images for: CAPE activates AMPK and Foxo3 signaling to induce growth inhibition and ferroptosis in triple-negative breast cancer
Source: PLoS One. 2024 Dec 27;19(12):e0315037. doi: 10.1371/journal.pone.0315037 (PMC11676562; doi:10.1371/journal.pone.0315037)

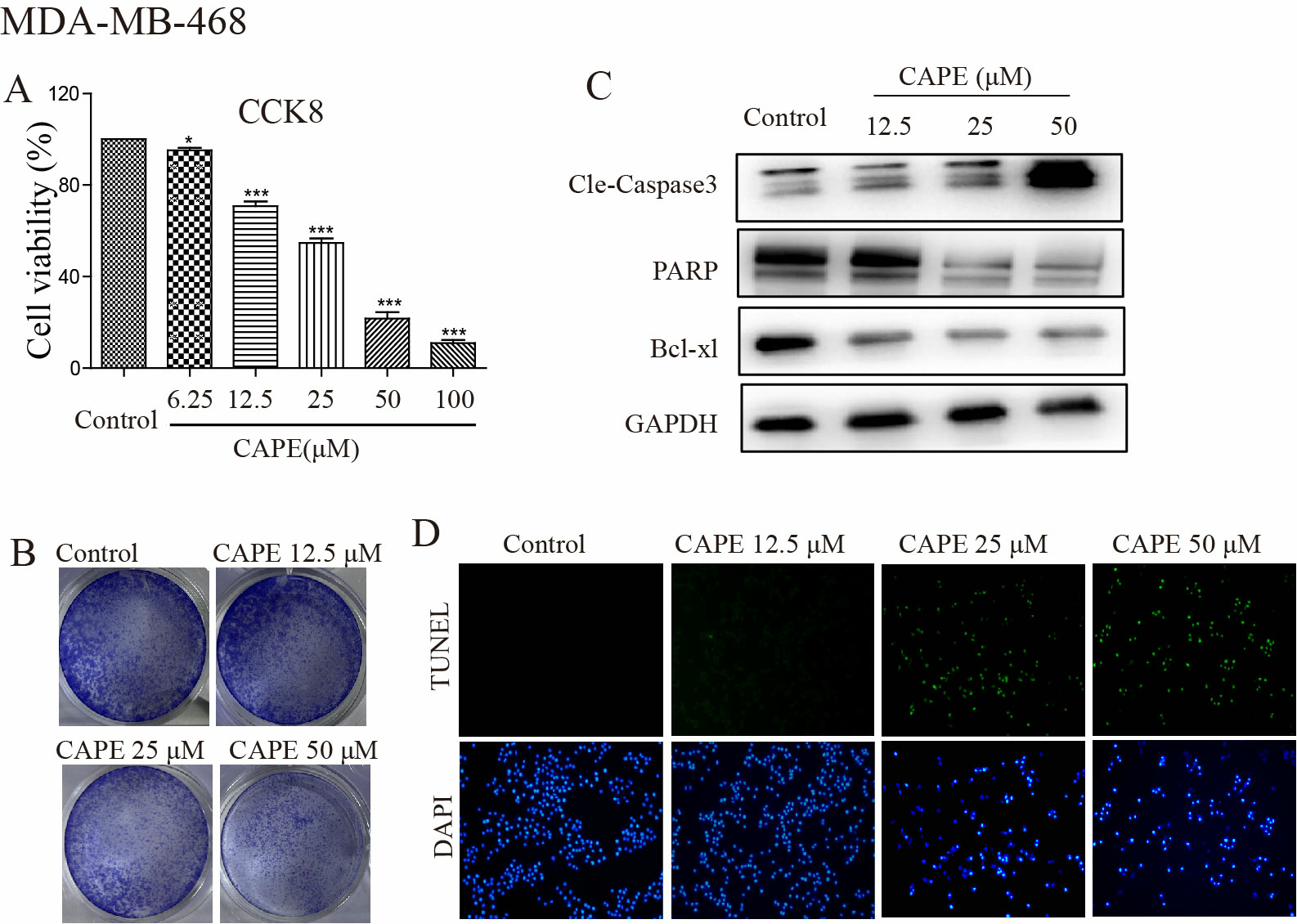

Supplement: S1 Fig — (A) MDA-MB-468 cells were treated with different concentrations of CAPE (6.25–100 μM) for 72 h, and CCK8 was used for cell viability detection. (B) MDA-MB-468 cell were treated with CAPE (12.5, 25, 50 μM) for 5 days to investigate cell colony formation. (C) MDA-MB-468 cells were treated with CAPE (12.5, 25, 50 μM) for 24 h, and western blot was used to detect the expression of Cle-Caspase3, PARP, and Bcl-xl. (D) MDA-MB-468 cells were treated with CAPE (12.5, 25, 50 μM) for 48 h, and the cell apoptosis was detected by TUNEL/DAPI dual staining. Values represent the mean ± SD from three independent experiments; *p <0.05, ***p<0.001: CAPE groups compared with the control group. (TIF) [file pone.0315037.s001.tif]

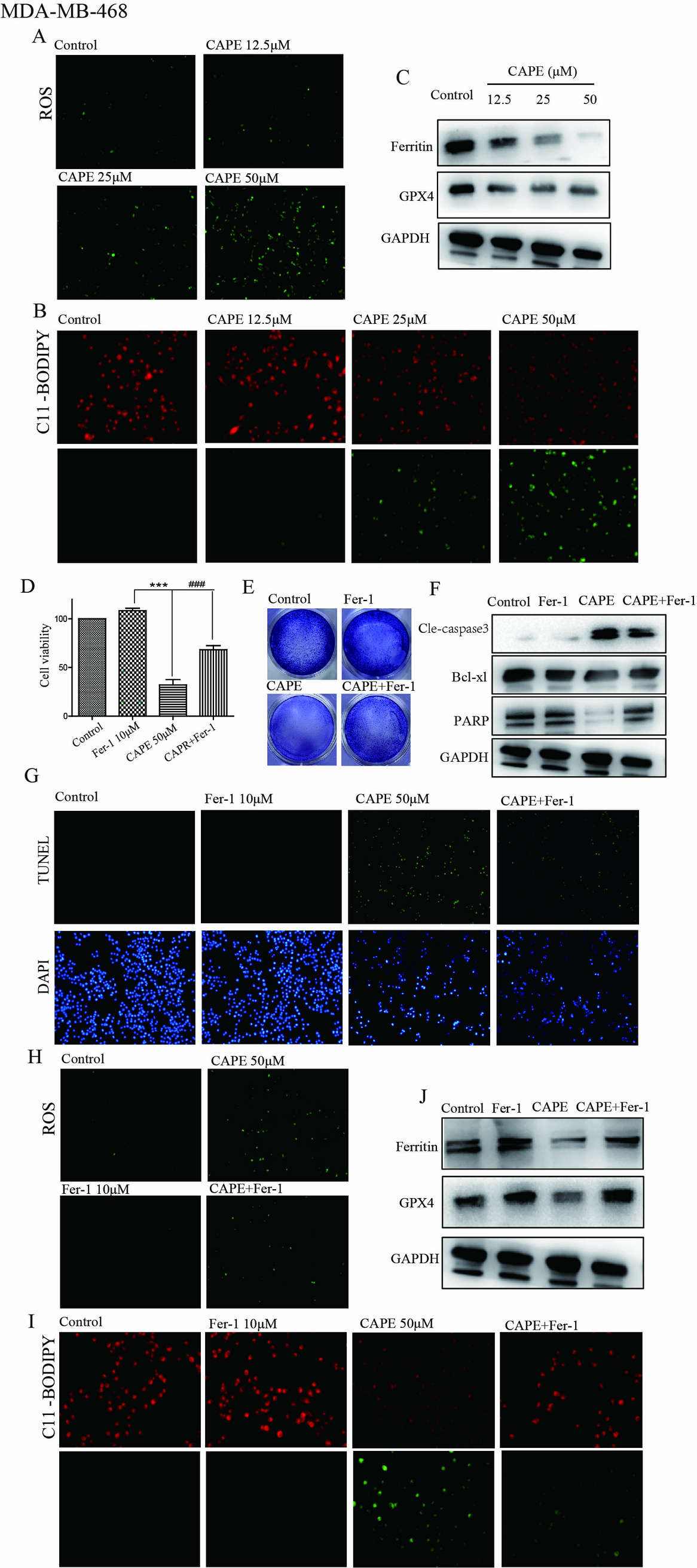

Supplement: S2 Fig — (A) Cells were treated with CAPE (12.5, 25, 50 μM) for 24 h, and DCFH-DA was used for ROS detection. (B) Cells were treated with CAPE (12.5, 25, 50 μM) for 24 h, and the lipid peroxidation was determined by C11-BODIPY staining. (C) Cells were treated with CAPE (12.5, 25, 50 μM) for 24 h, and western blot was used to detect the expression of GPX4 and Ferritin. (D-G) Cells were treated singly or in combination with Fer-1 (10 μM) and CAPE (50 μM), and cell viability was determined by CCK8 at 72 h. colony formation assay was performed after 5 days. TUNEL staining and western blot were conducted for apoptotic cells and apoptosis-related proteins at 48 h and 24 h. (H-J) Cells were treated with singly or in combination with Fer-1 (10 μM) and CAPE (50 μM) for 24 h, and the lipid peroxidation was determined by ROS and C11-BODIPY staining, along with GPX4 and Ferritin expression by western blot. Values represent the mean ± SD from three independent experiments. ***p<0.001: CAPE groups compared with the control group; ###p<0.001: CAPE+Fer-1 groups compared with the CAPE group. (TIF) [file pone.0315037.s002.tif]

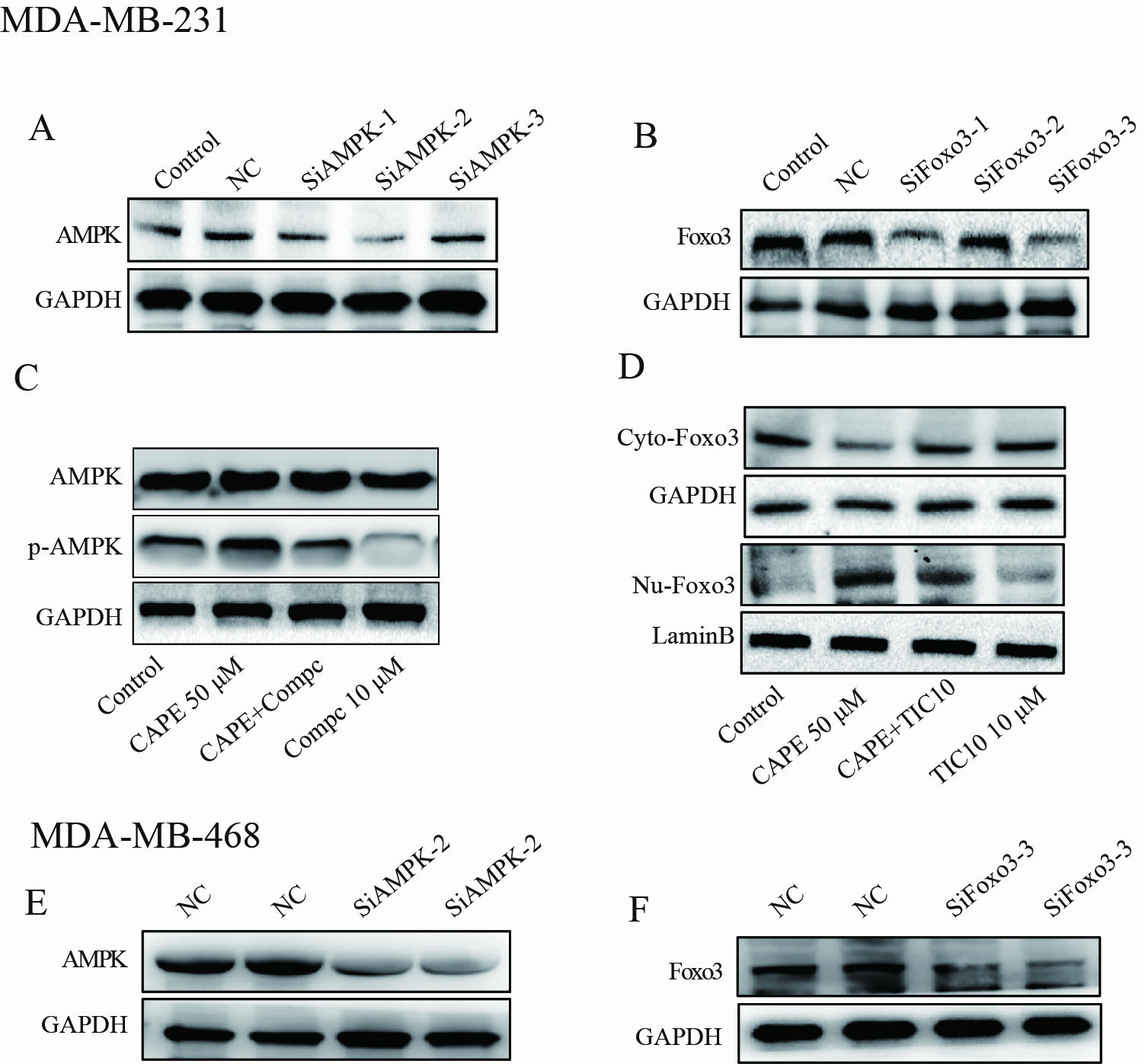

Supplement: S3 Fig — (A-B) MDA-MB-231 cells were transfected with small interfering RNA targeting AMPK or Foxo3, and western blot was used to detect transfection efficiency. (C) MDA-MB-231 cells were treated singly or in combination with Compc (10 μM) and CAPE (50 μM) for 12 h, and the protein levels of p-AMPK and GAPDH were analyzed by western blot. (D) MDA-MB-231 cells were treated singly or in combination with TIC10 (10 μM) and CAPE (50 μM) for 12 h. The protein level of Foxo3 in cytoplasm and nuclear were analyzed by western blot. (E-F) MDA-MB-468 cells were transfected with small interfering RNA targeting AMPK or Foxo3, and western blot was used to detect transfection efficiency. Values represent the mean ± SD from three independent experiments. (TIF) [file pone.0315037.s003.tif]

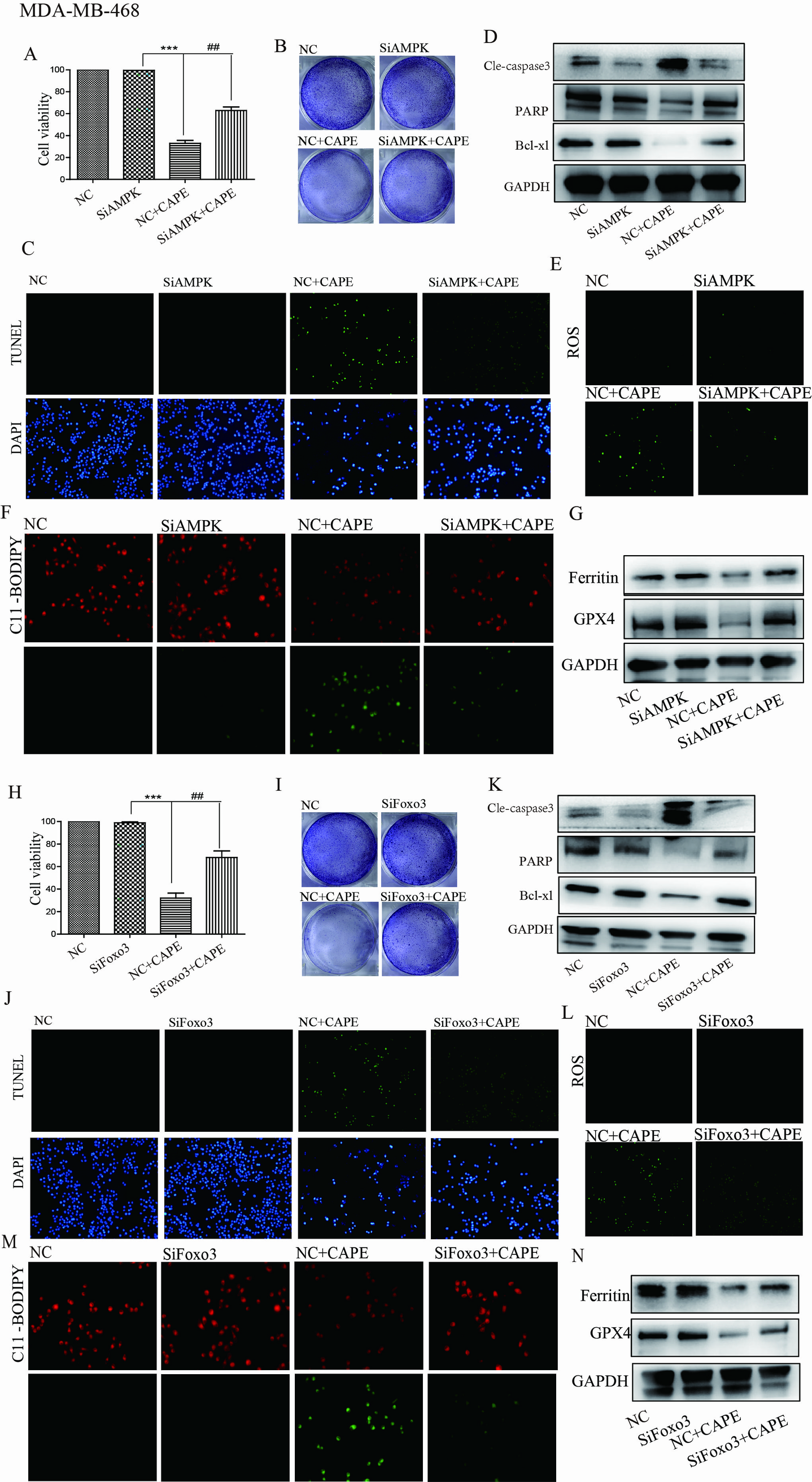

Supplement: S4 Fig — (A-D) Cells were transfected with siRNA targeting AMPK. The CCK8 was used for the cell viability detection at 72 h. The colony was investigated by colony formation assay after 5 days. The cell apoptosis was detected by TUNEL/DAPI dual staining at 48 h. The expression of Cle-caspase3, PARP, and Bcl-xl were detected by western blot at 24 h. (E-G) Cells were transfected with siRNAs targeting AMPK for 24 h, DCFH-DA was used for ROS detection, C11-BODIPY staining was performed for lipid peroxidation detection and western blot was used for GPX4 and Ferritin levels test. (H-K) Cells were transfected with siRNA targeting Foxo3. The CCK8 was used for the cell viability detection at 72 h. The colony was investigated by colony formation assay after 5 days. The cell apoptosis was detected by TUNEL/DAPI dual staining at 48 h. The expression of Cle-caspase3, PARP, and Bcl-xl were detected by western blot at 24 h. (L-N) Cells were transfected with siRNAs targeting Foxo3 for 24 h, DCFH-DA was used for ROS detection, C11-BODIPY staining was performed for lipid peroxidation detection and western blot was used for GPX4 and Ferritin levels test. Values represent the mean ± SD from three independent experiments. ***p<0.001: the SiAMPK or SiFoxo3 groups compared with the NC+CAPE groups; ##p<0.01: the SiAMPK+CAPE or SiFoxo3+CAPE groups compared with the NC+CAPE groups. (TIF) [file pone.0315037.s004.tif]

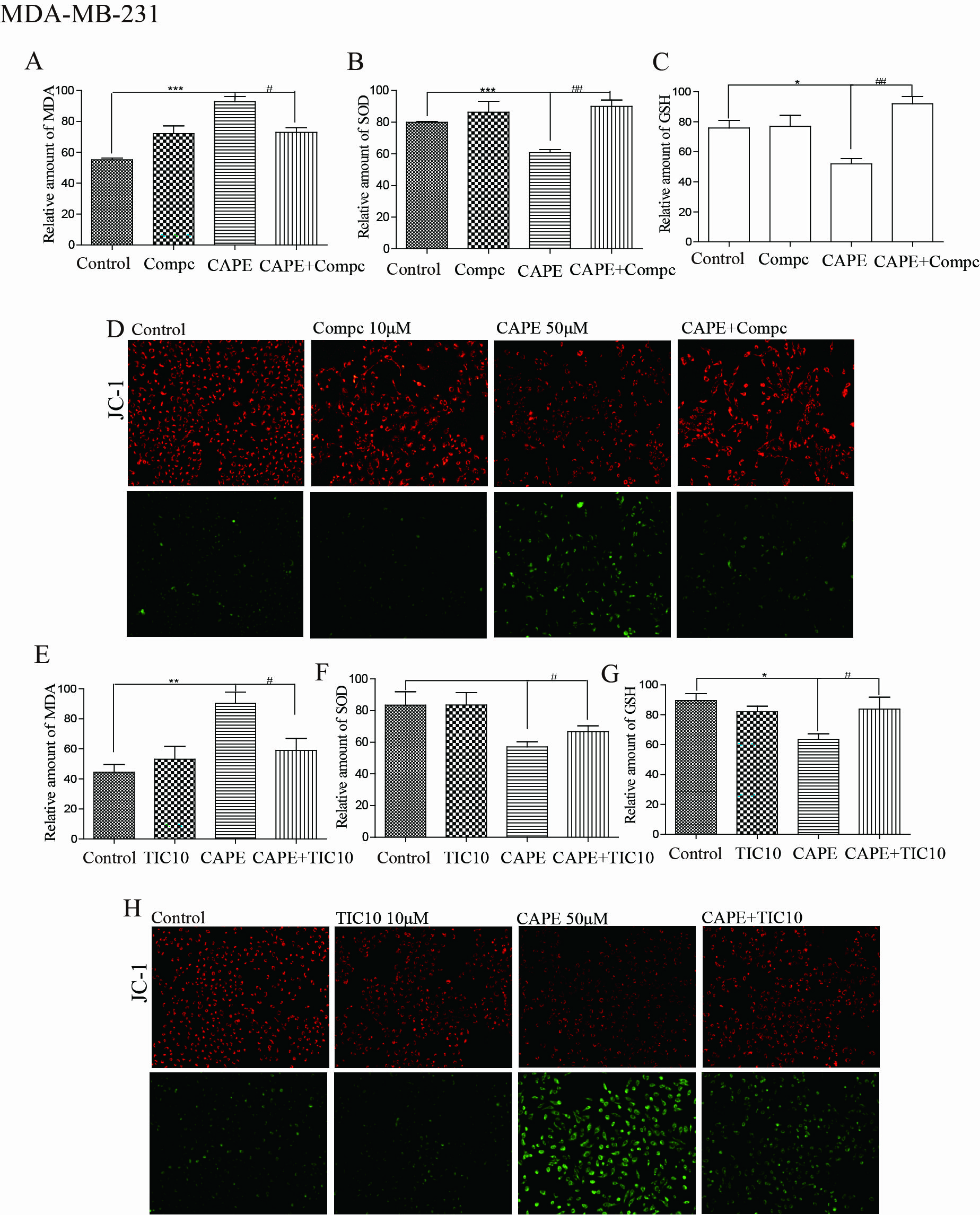

Supplement: S5 Fig — (A-C) Cells were treated singly or in combination with Compc (10 μM) and CAPE (50 μM) for 24 h to investigate ROS, MDA, SOD and GSH levels. (D) Cells were treated singly or in combination with Compc (10 μM) and CAPE (50 μM) for 24 h, and JC-1 staining was used to detect the mitochondrial membrane potential. (E-G) Cells were treated singly or in combination with TIC10 (10 μM) and CAPE (50 μM) for 24 h to investigate ROS, MDA, SOD and GSH levels. (H) Cells were treated singly or in combination with TIC10 (10 μM) and CAPE (50 μM) for 24 h, and JC-1 staining was used to detect the mitochondrial membrane potential. Values represent the mean ± SD from three independent experiments. *p<0.05, **p<0.01***p<0.001: CAPE compared with control, #p <0.05, ##p<0.01: combinatorial Compc+CAPE or TIC10+CAPE groups compared with the CAPE group. (TIF) [file pone.0315037.s005.tif]
