# Supplementary material for: CAPE activates AMPK and Foxo3 signaling to induce growth inhibition and ferroptosis in triple-negative breast cancer
Source: PLoS One. 2024 Dec 27;19(12):e0315037. doi: 10.1371/journal.pone.0315037 (PMC11676562; doi:10.1371/journal.pone.0315037)
Supplement: S1 Raw images — (PDF) [file pone.0315037.s006.pdf]

Figure1

C

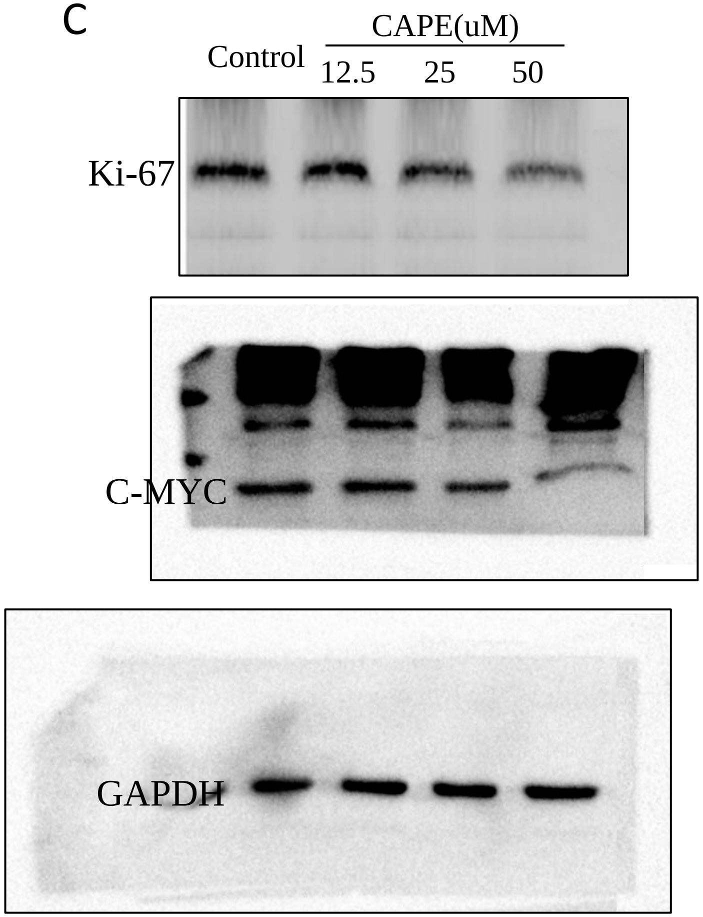
CAPE(uM)
Control
12.5
25
50
Ki-67
C-MYC
GAPDH

F

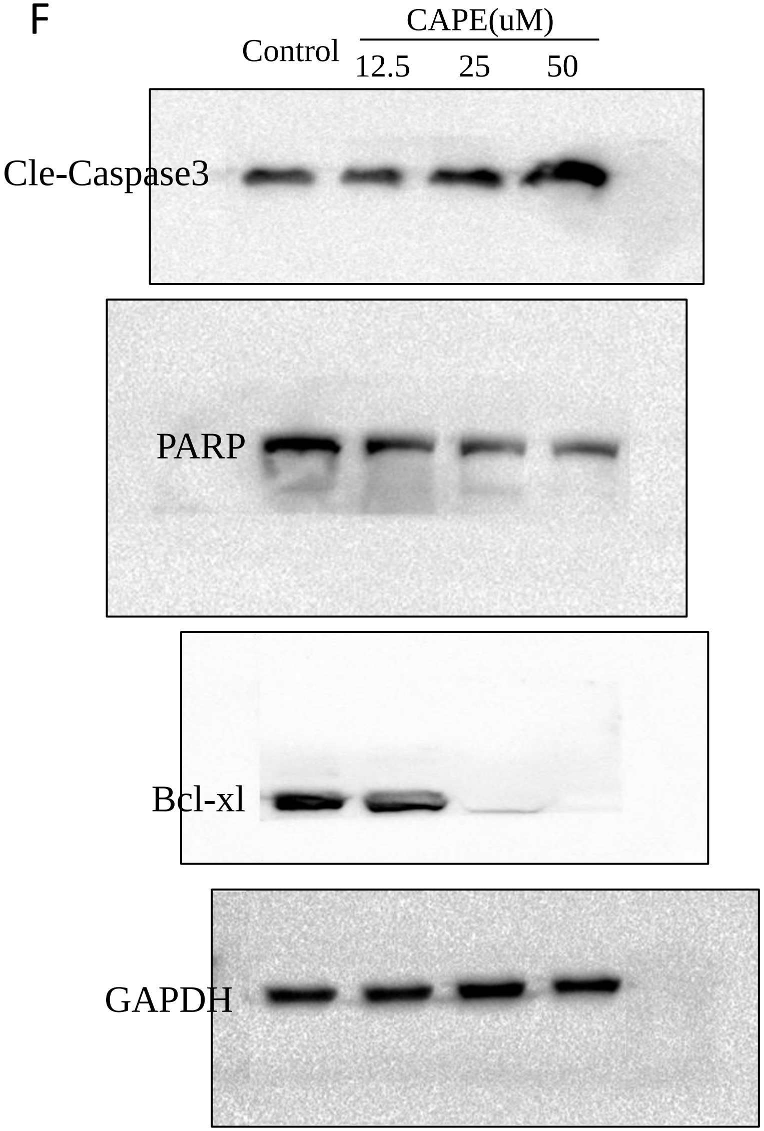
CAPE(uM)
Control
12.5
25
50
Cle-Caspase3
PARP
Bcl-xl
GAPDH

Figure2

G

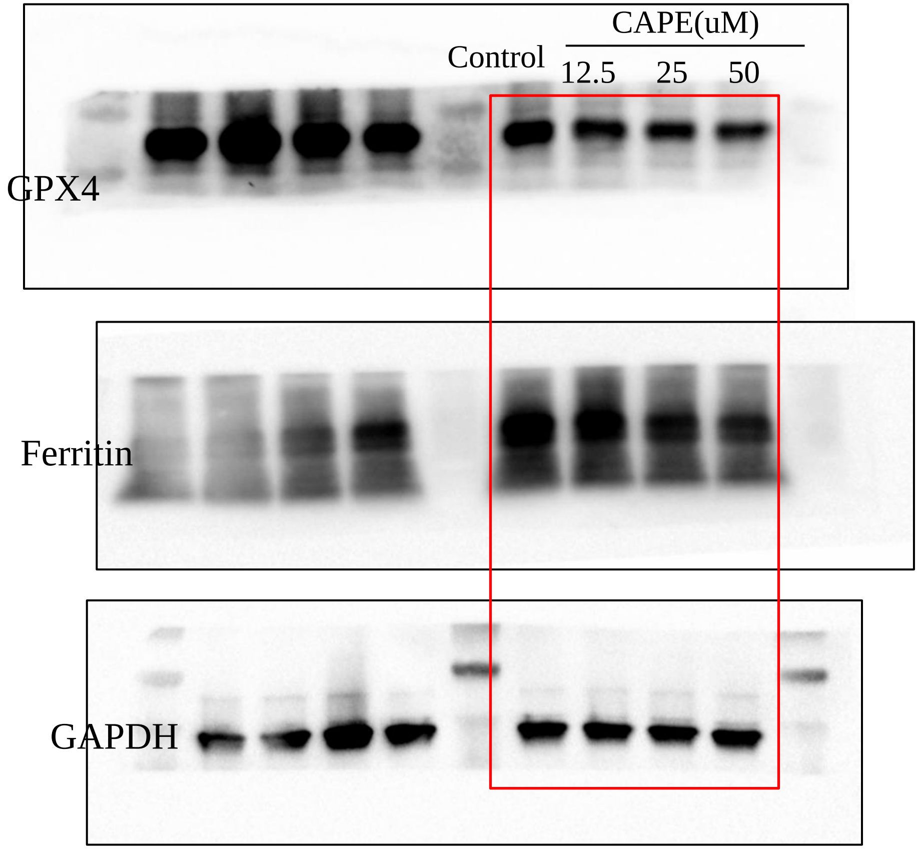

Figure3

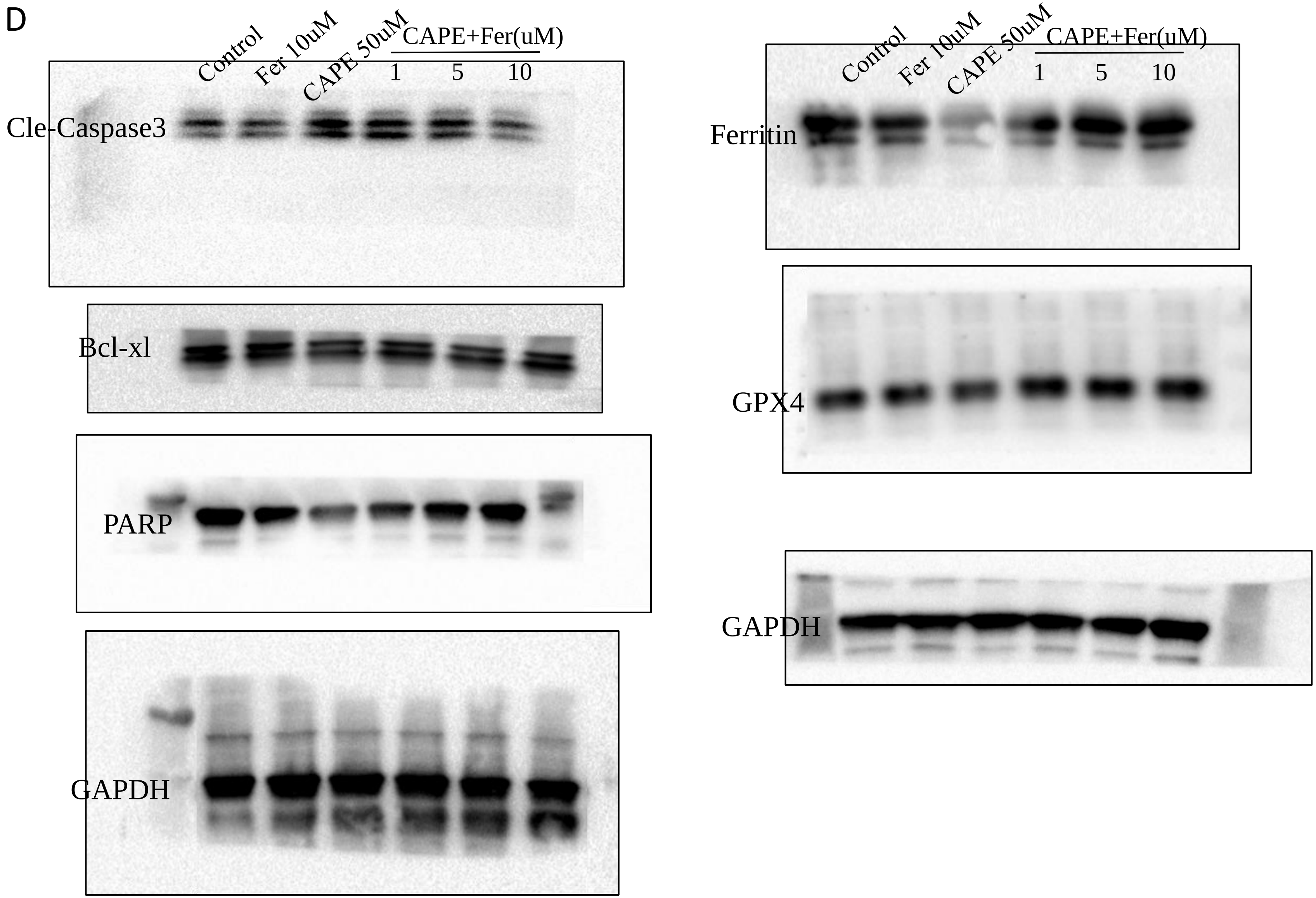
D
Control
Fer 10uM
CAPE 50uM
CAPE+Fer(uM)
1
5
10
Cle-Caspase3
Bcl-xl
PARP
GAPDH
Ferritin
GPX4
GAPDH

Figure4

A

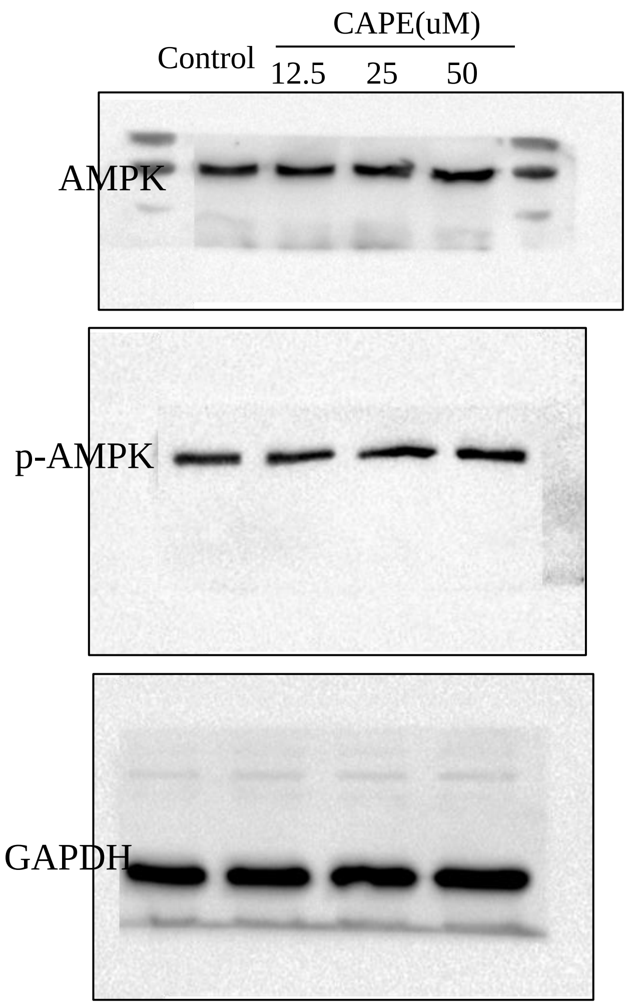

Control
CAPE(uM)
12.5
25
50
AMPK
p-AMPK
GAPDH

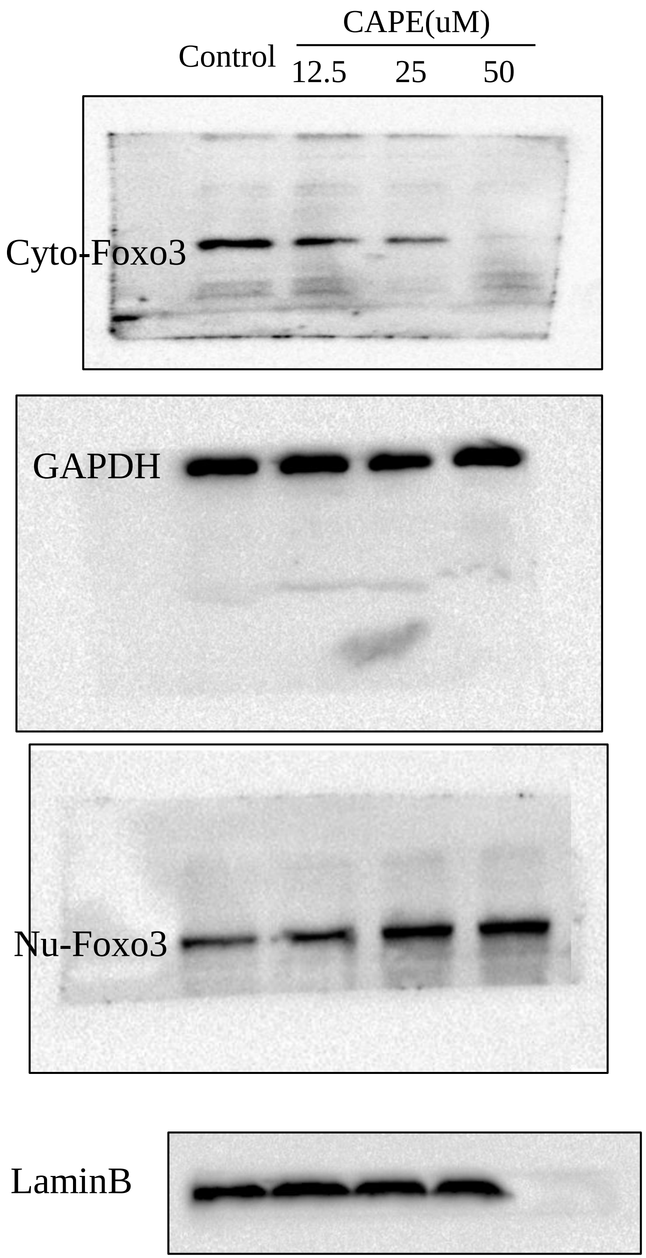

Control
CAPE(uM)
12.5
25
50
Cyto-Foxo3
GAPDH
Nu-Foxo3
LaminB

Figure4

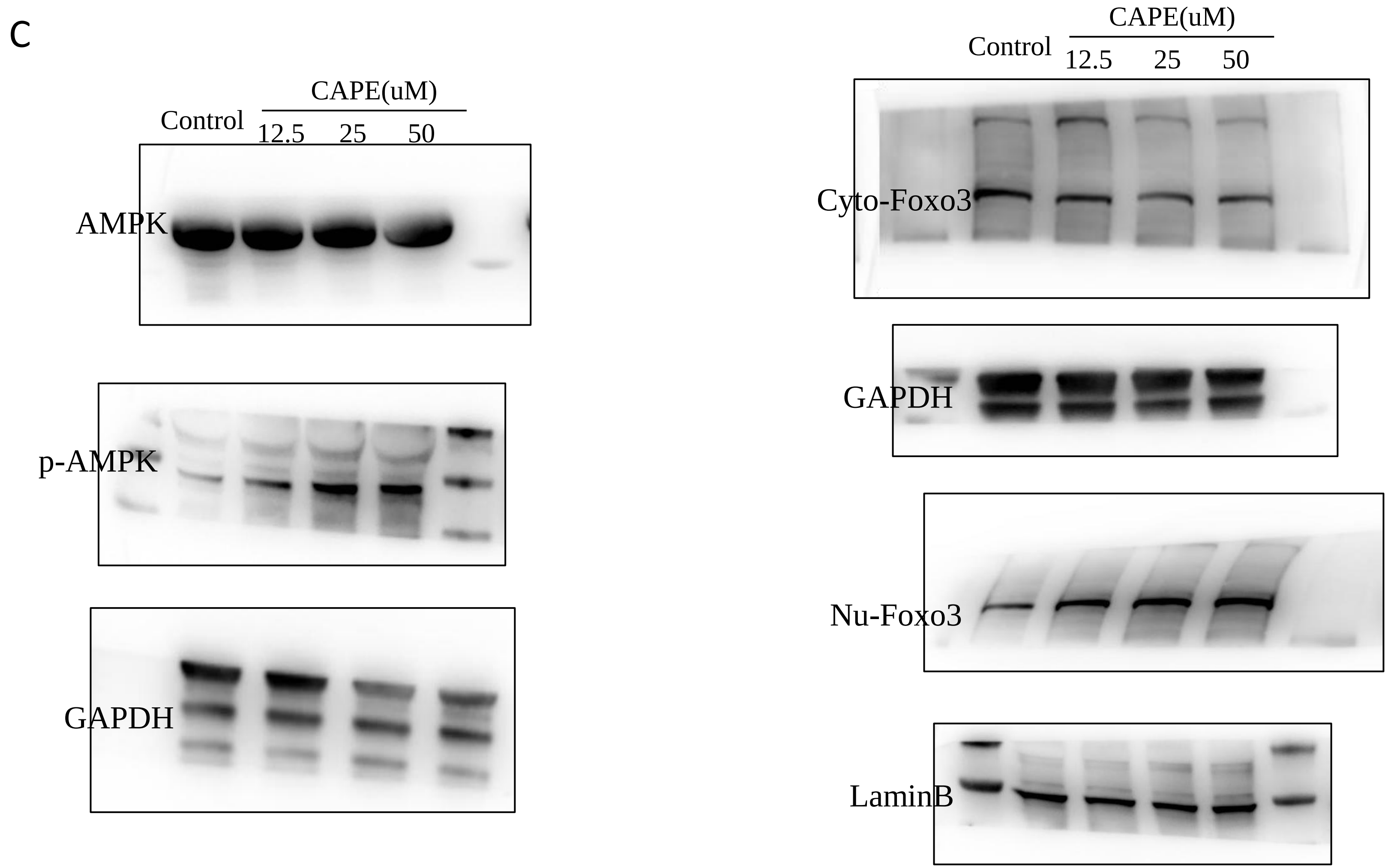
C
CAPE(uM)
Control
12.5
25
50
AMPK
p-AMPK
GAPDH
CAPE(uM)
Control
12.5
25
50
Cyto-Foxo3
GAPDH
Nu-Foxo3
LaminB

Figure5

C

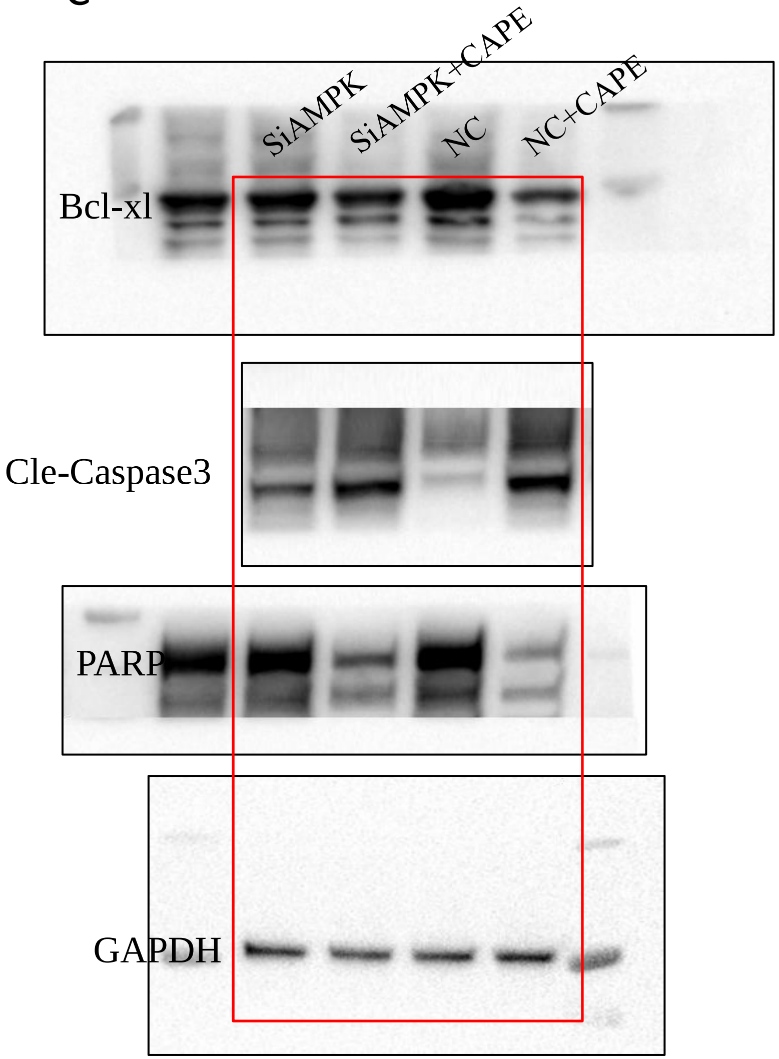
SiAMPK
SiAMPK+CAPE
NC
NC+CAPE
Bcl-xl
Cle-Caspase3
PARP
GAPDH

F

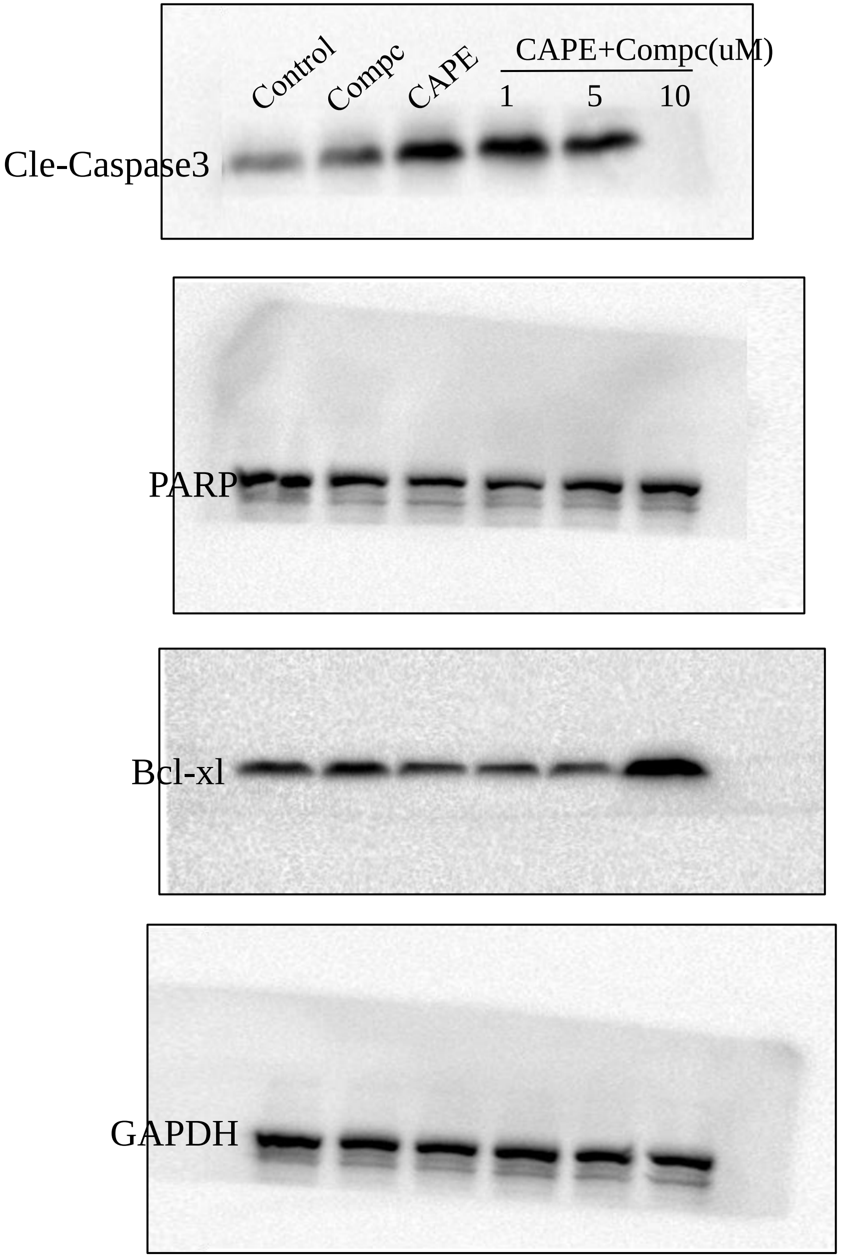
Control
Compc
CAPE
CAPE+Compc(uM)
1
5
10
Cle-Caspase3
PARP
Bcl-xl
GAPDH

Figure5

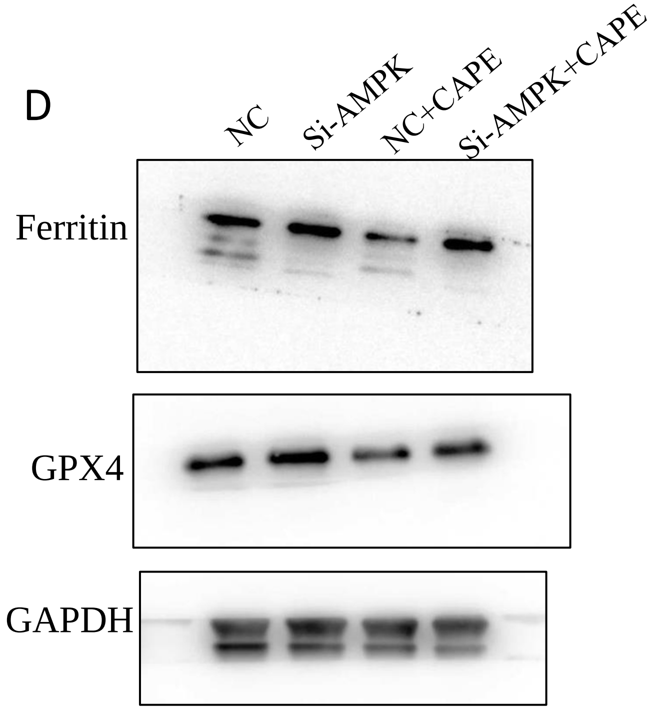
D
NC
Si-AMPK
NC+CAPE
Si-AMPK+CAPE
Ferritin
GPX4
GAPDH

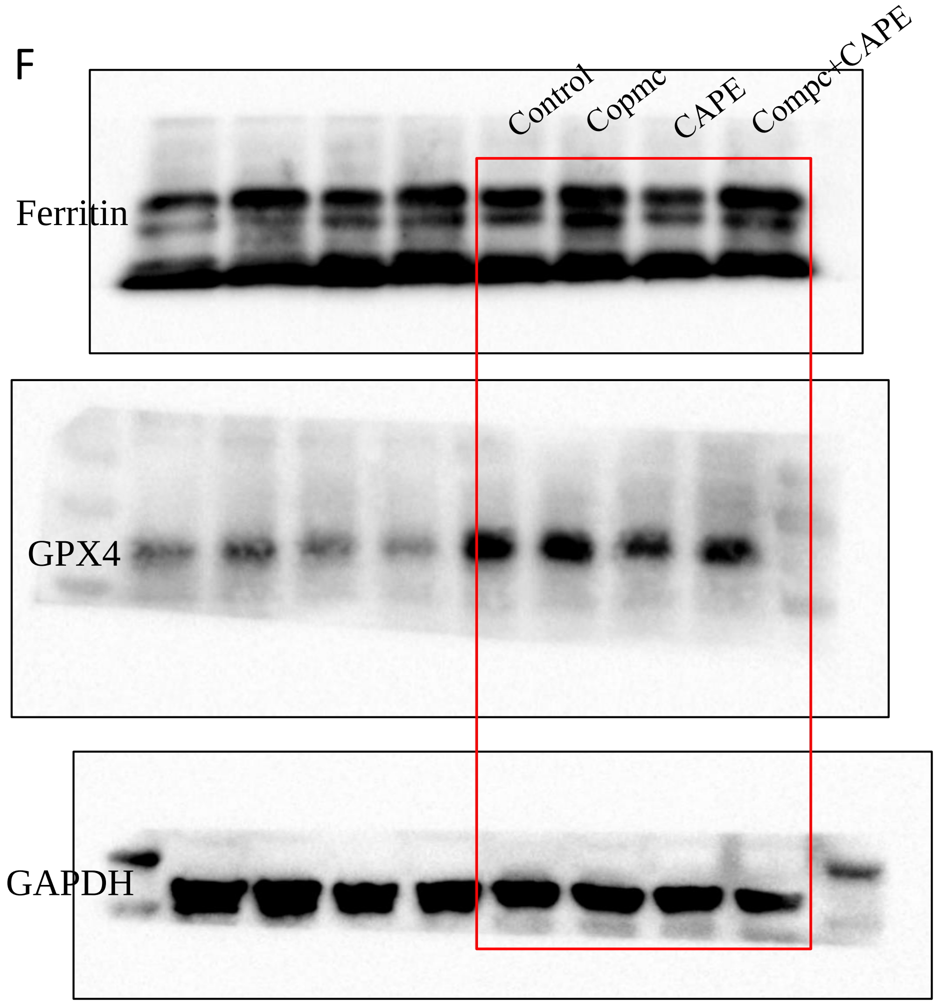
F
Control
Copmc
CAPE
Compc+CAPE
Ferritin
GPX4
GAPDH

Figure6

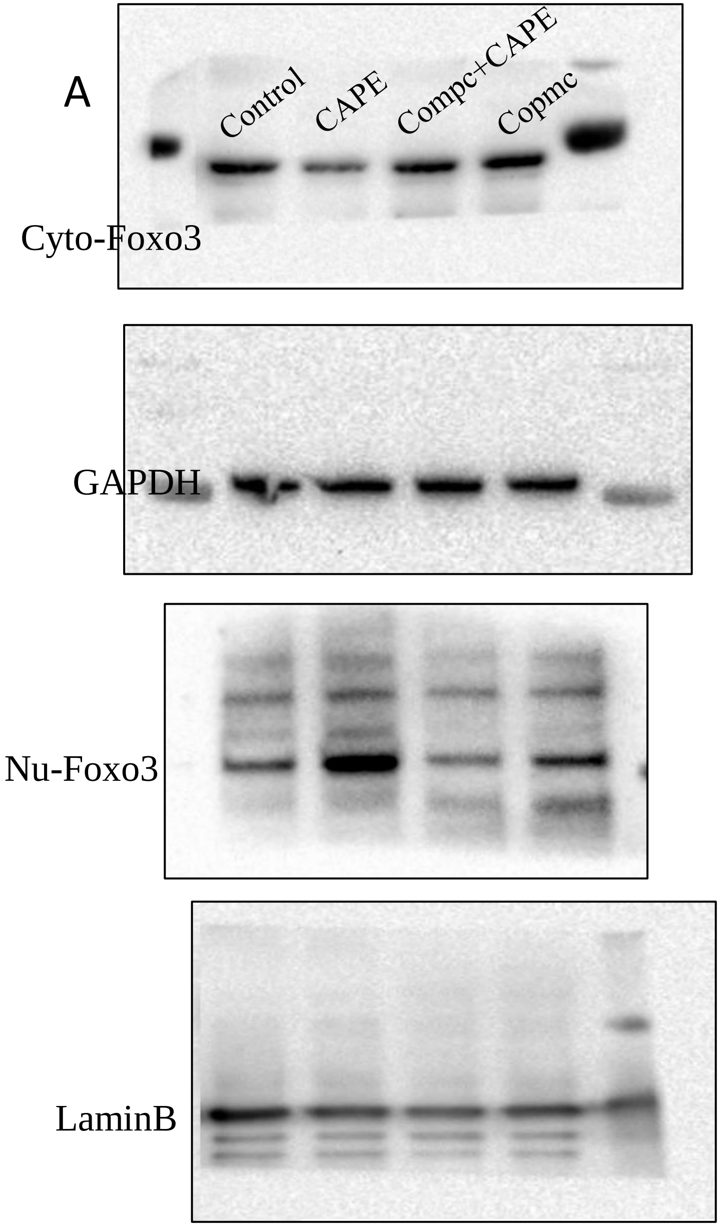
A
Control
CAPE
Compc+CAPE
Copmc
Cyto-Foxo3
GAPDH
Nu-Foxo3
LaminB

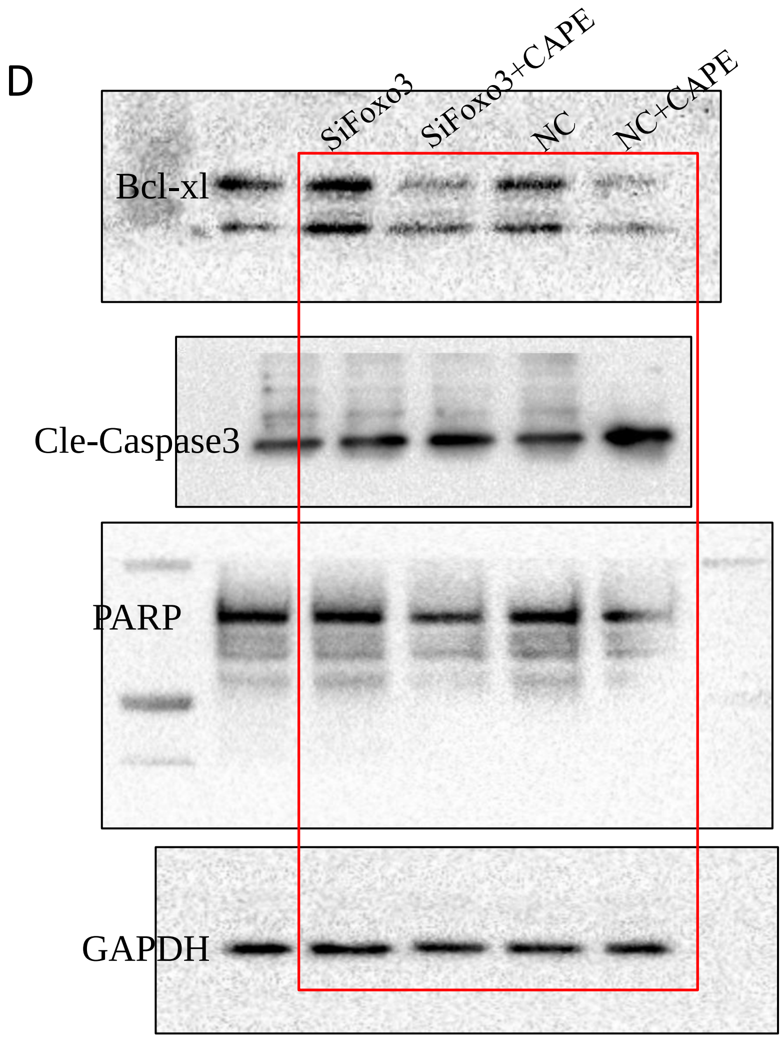
D
SiFoxo3
SiFoxo3+CAPE
NC
NC+CAPE
Bcl-xl
Cle-Caspase3
PARP
GAPDH

Figure6

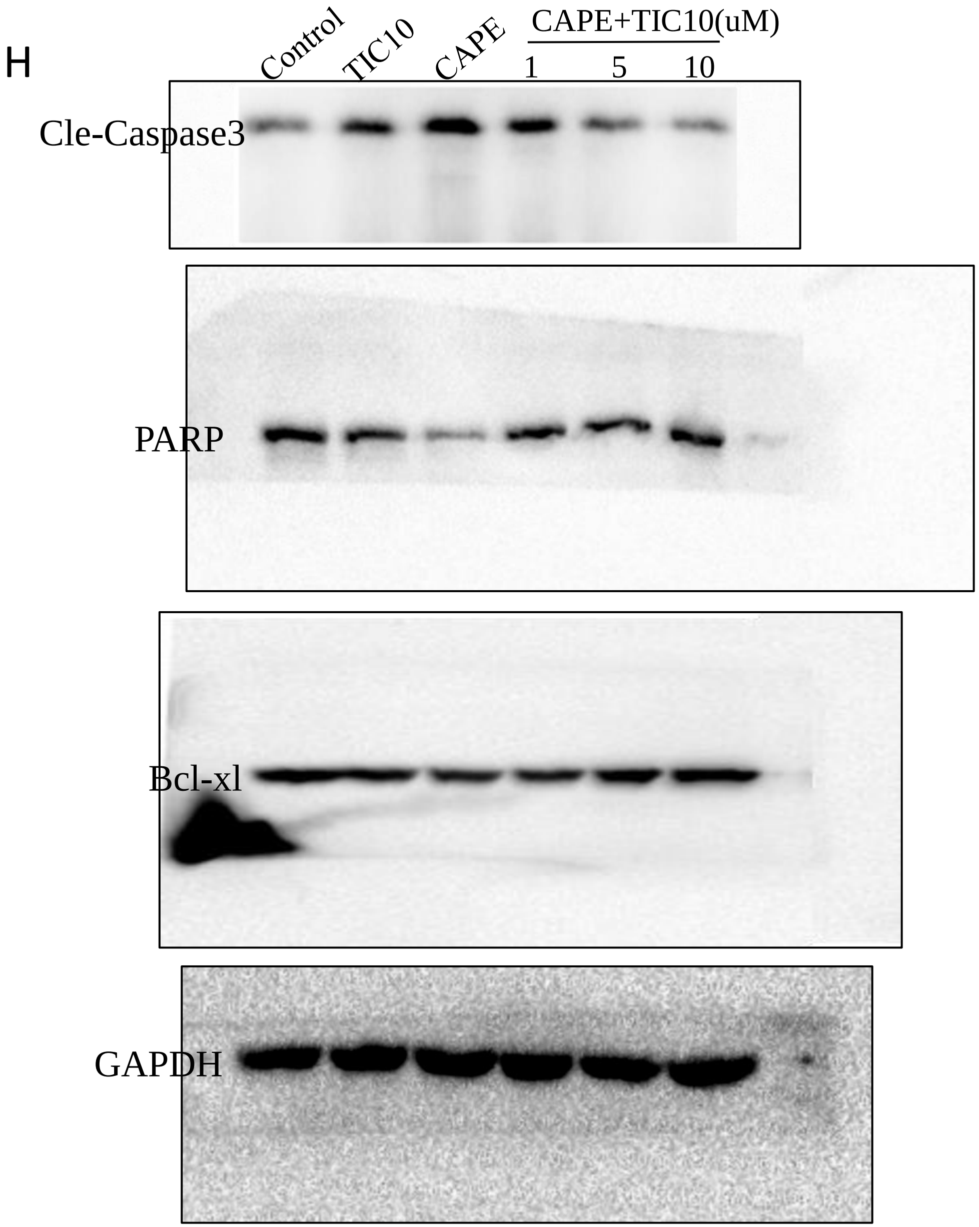
H
Control
TIC10
CAPE
CAPE+TIC10(uM)
1
5
10
Cle-Caspase3
PARP
Bcl-xl
GAPDH

Figure6

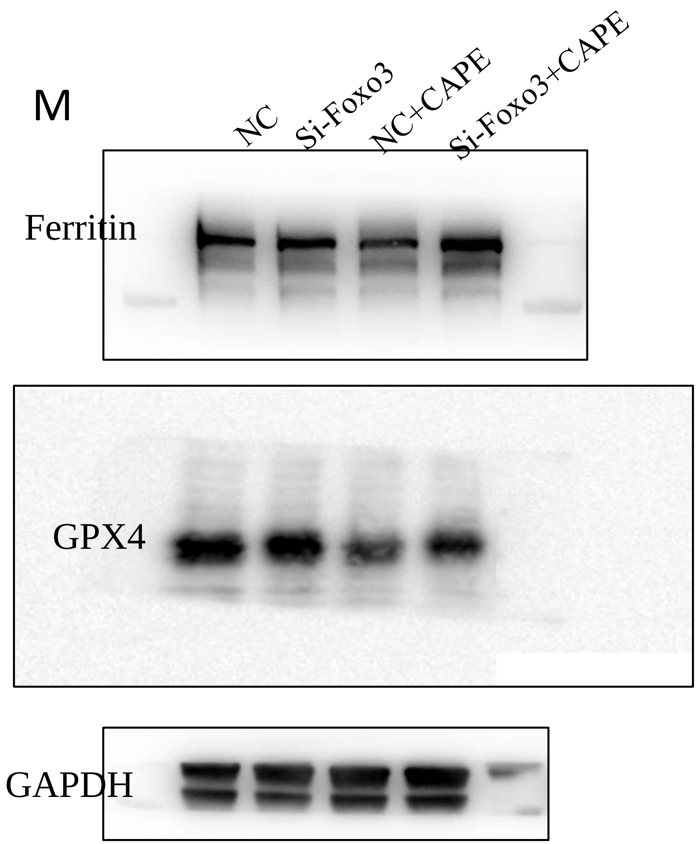
M
NC
Si-Foxo3
NC+CAPE
Si-Foxo3+CAPE
Ferritin
GPX4
GAPDH

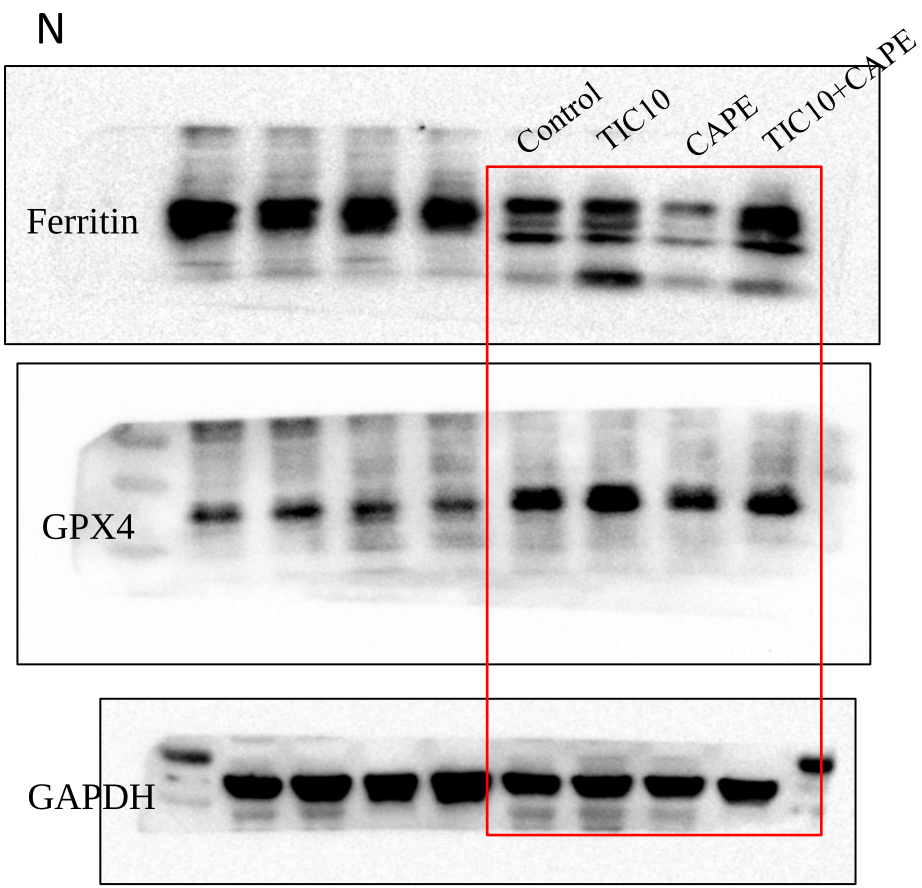
N
Control
TIC10
CAPE
TIC10+CAPE
Ferritin
GPX4
GAPDH

Figure7

E

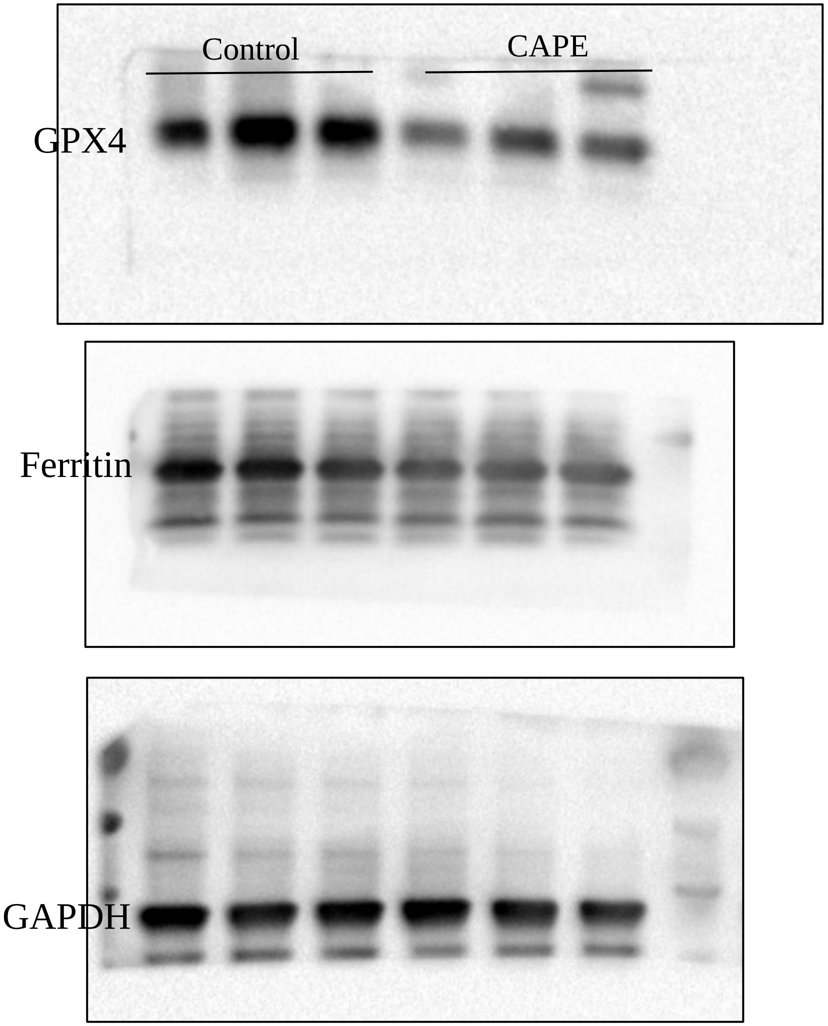

SupFigure1

C

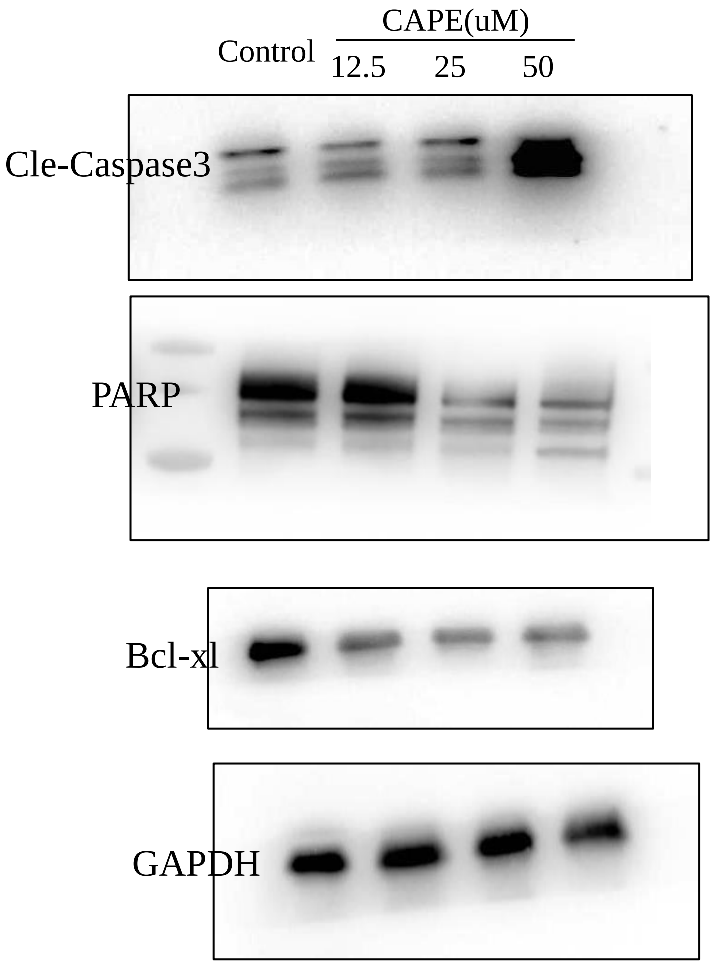

SupFigure2

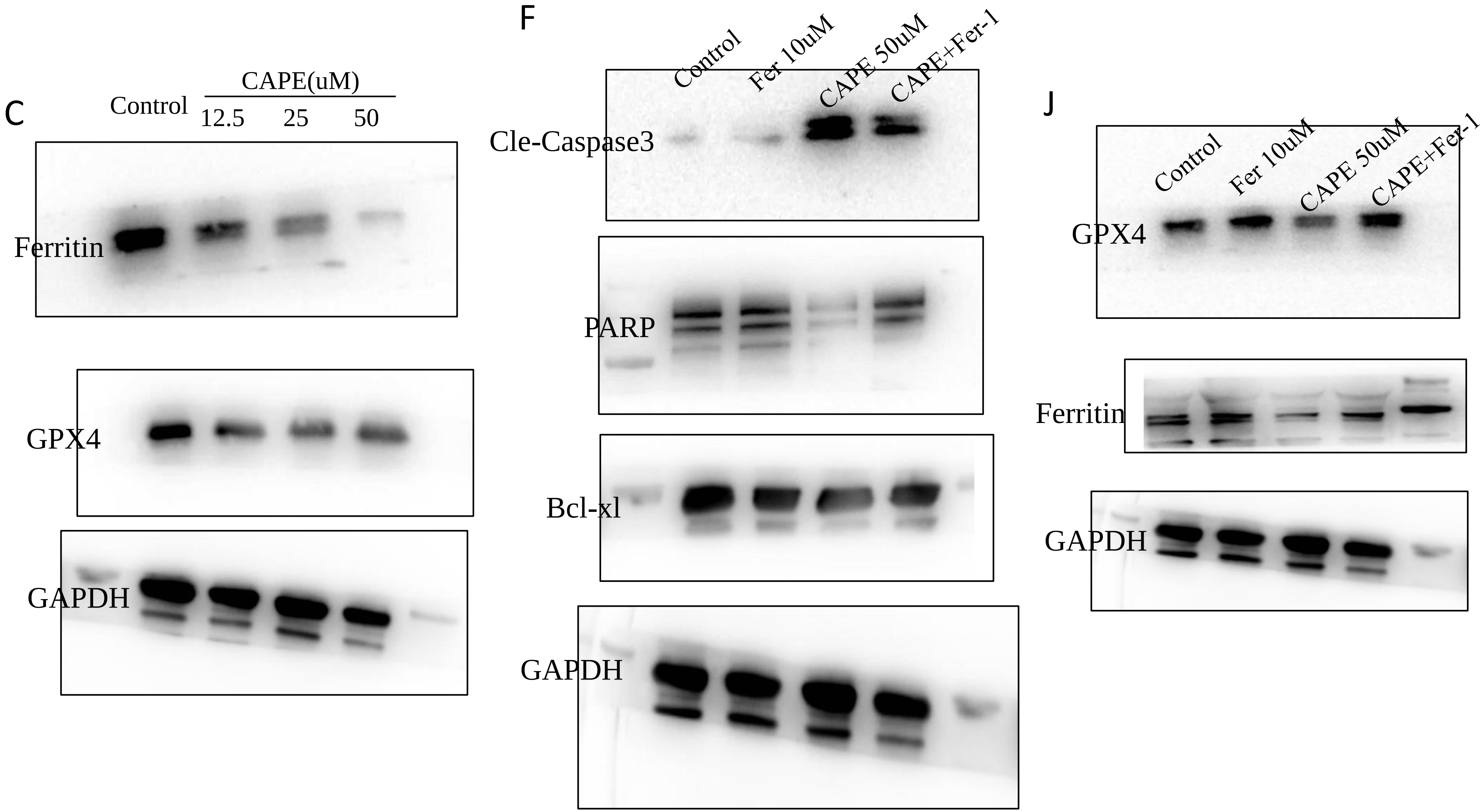
C
Control
CAPE(uM)
12.5
25
50
Ferritin
GPX4
GAPDH
F
Control
Fer 10uM
CAPE 50uM
CAPE+Fer-1
Cle-Caspase3
PARP
Bcl-xl
GAPDH
J
Control
Fer 10uM
CAPE 50uM
CAPE+Fer-1
GPX4
Ferritin
GAPDH

SupFigure3

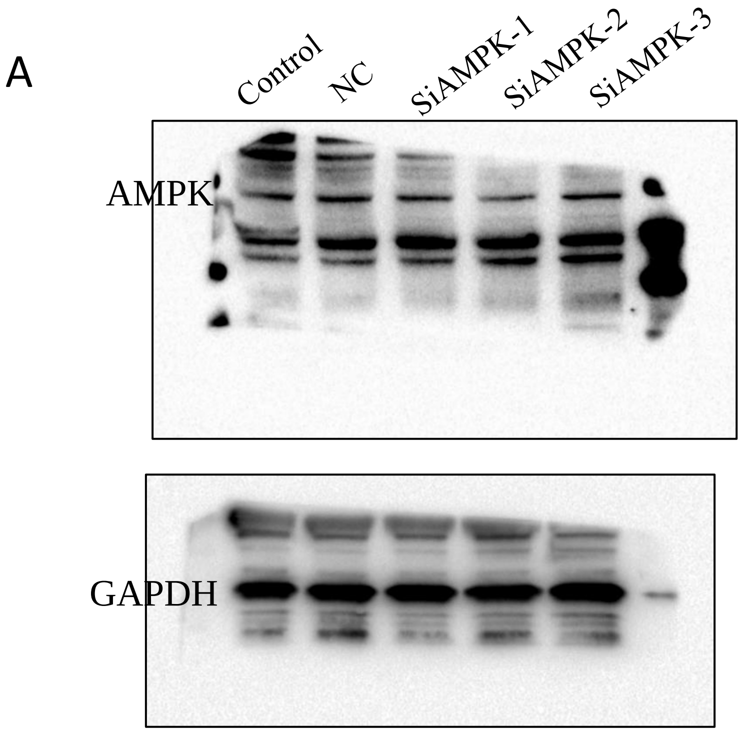
A
Control
NC
SiAMPK-1
SiAMPK-2
SiAMPK-3
AMPK
GAPDH

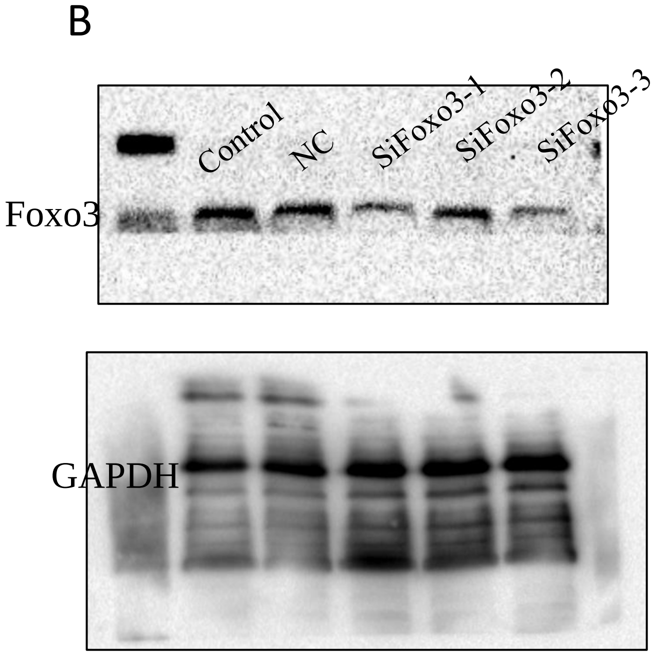
B
Control
NC
SiFoxo3-1
SiFoxo3-2
SiFoxo3-3
Foxo3
GAPDH

SupFigure3

C

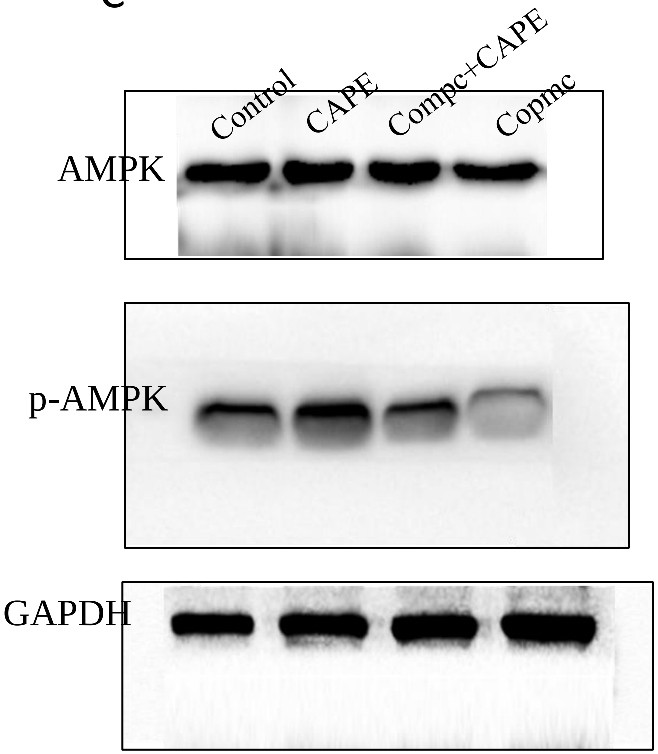

D

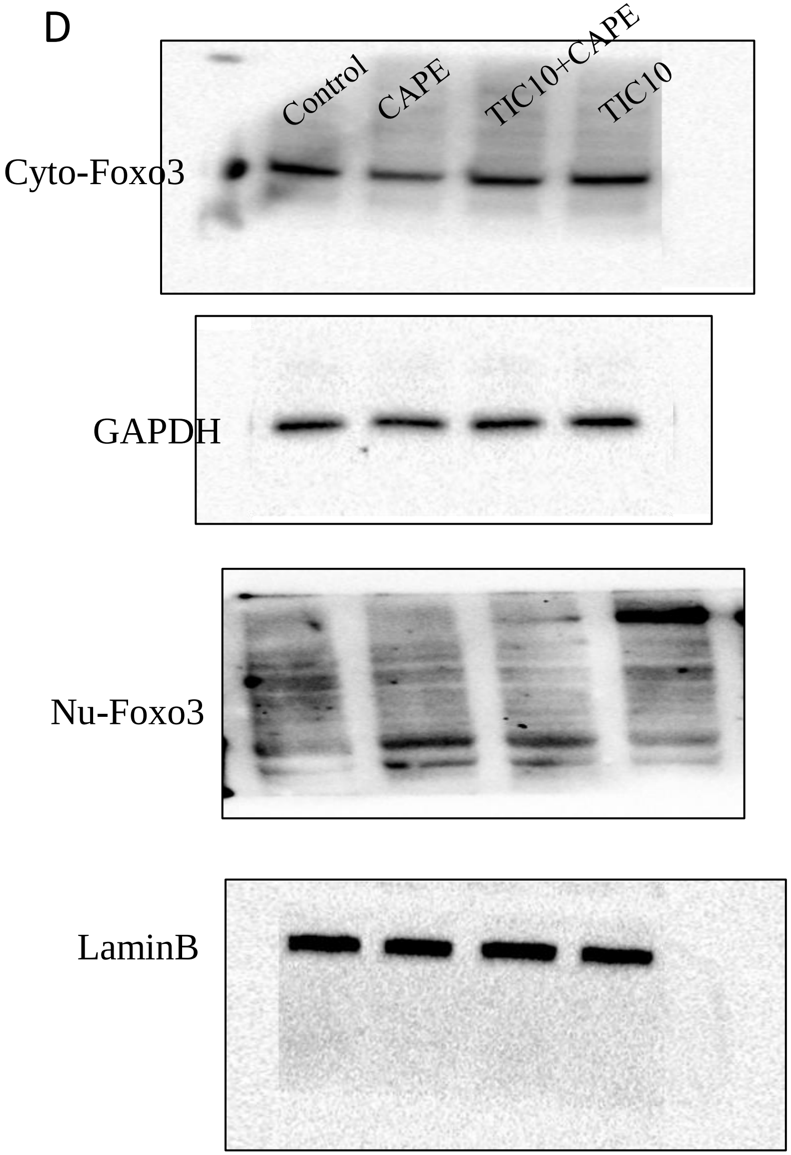

SupFigure3

E

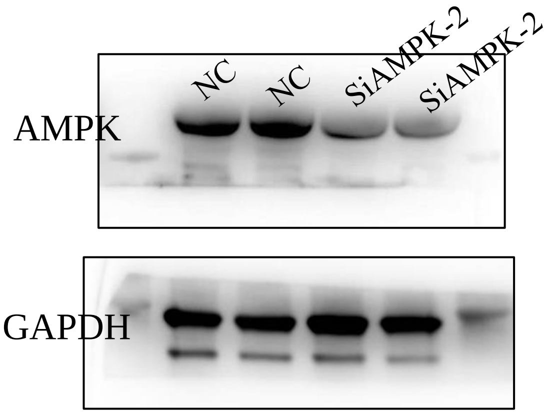

F

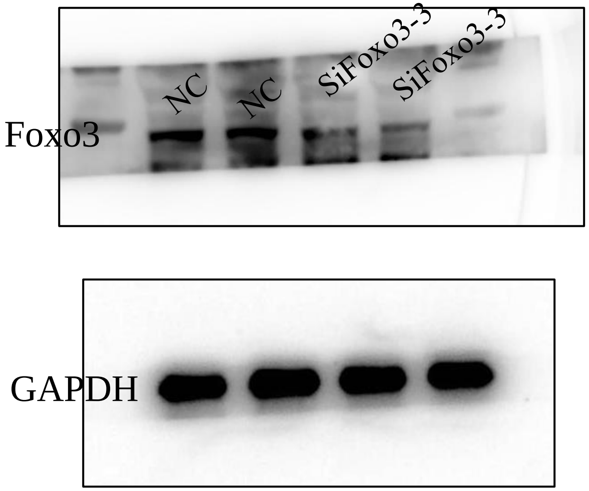

SupFigure5

F

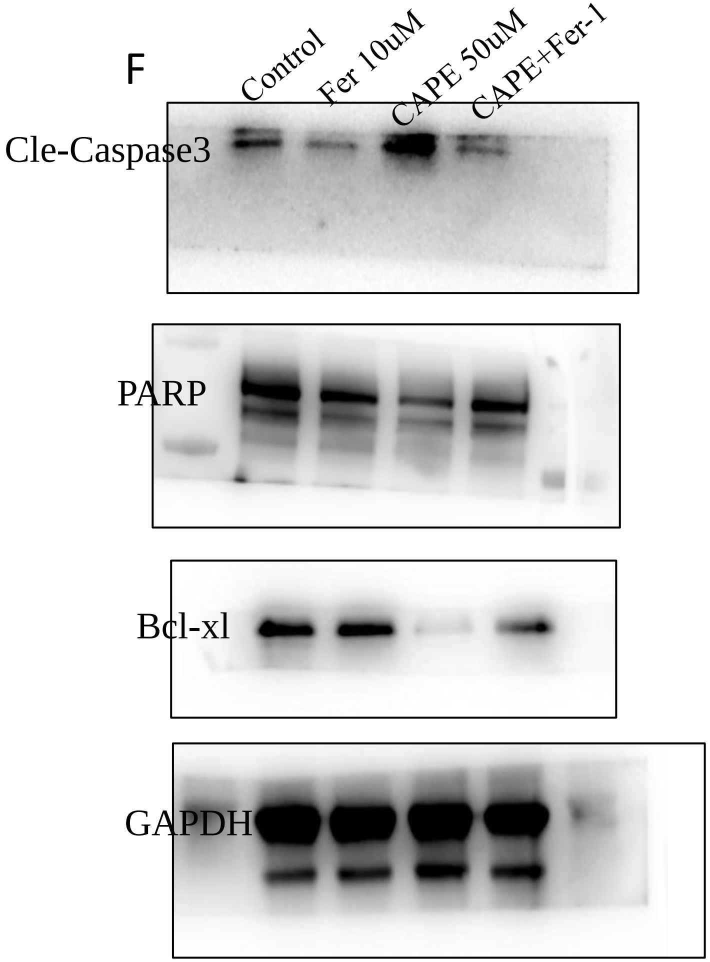

G

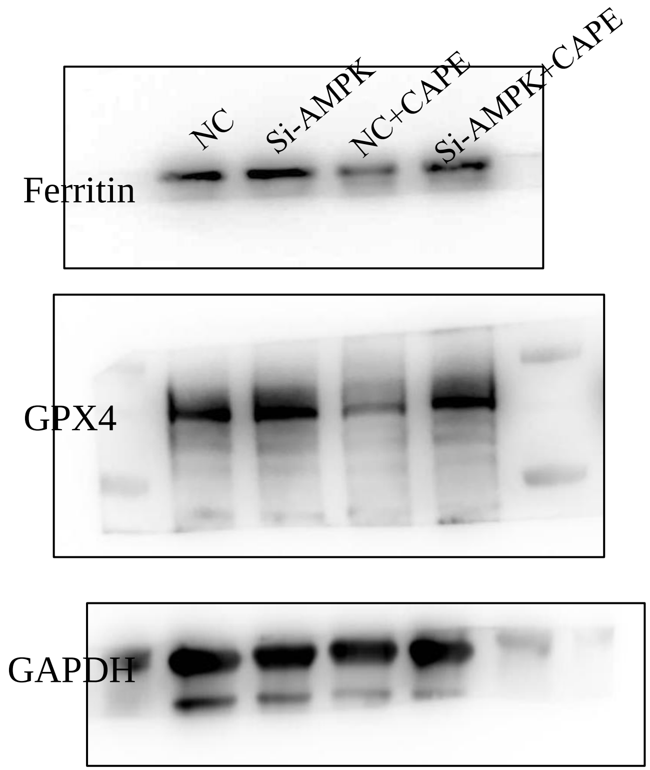

SupFigure5

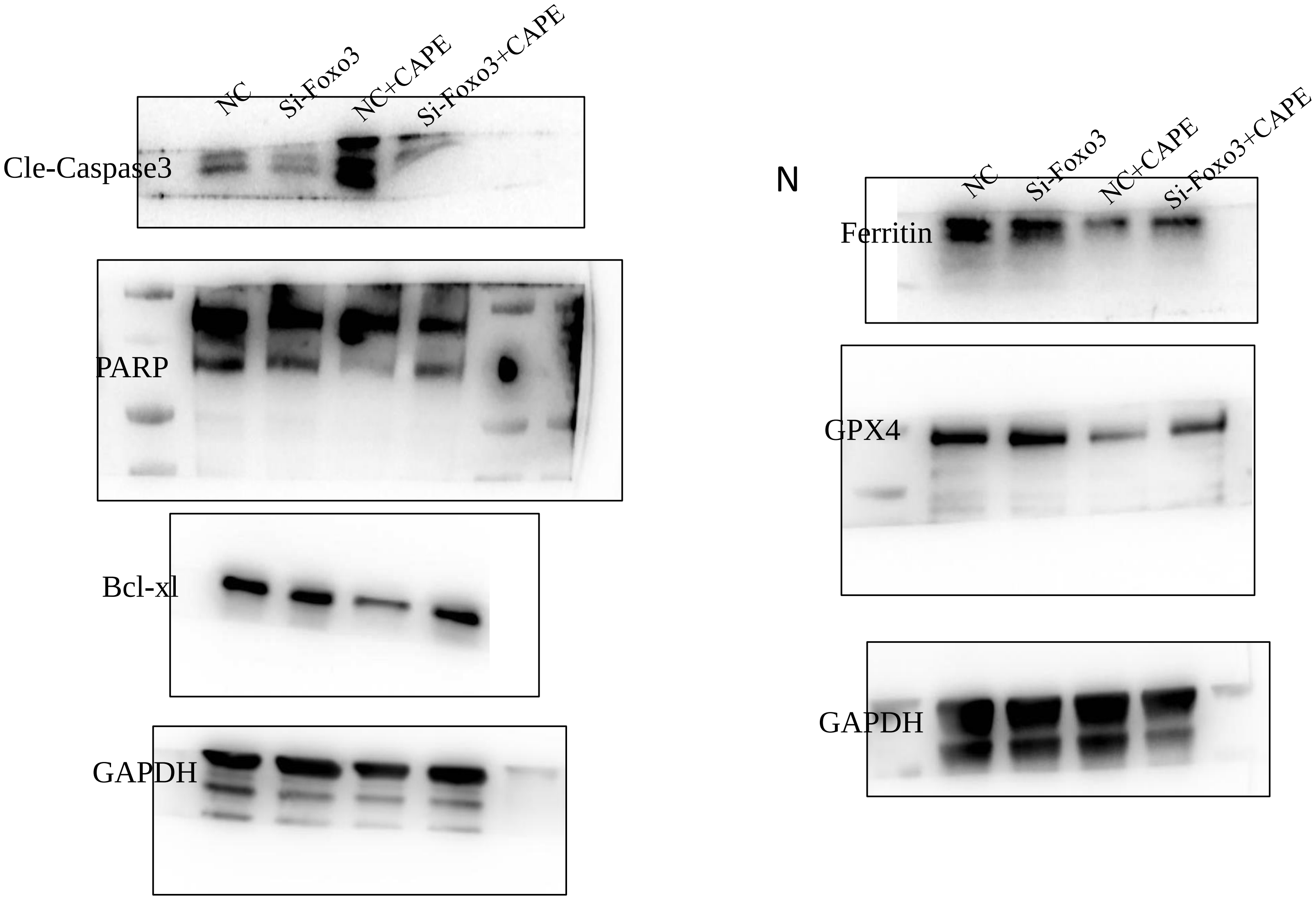
NC
Si-Foxo3
NC+CAPE
Si-Foxo3+CAPE
Cle-Caspase3
PARP
Bcl-xl
GAPDH
N
NC
Si-Foxo3
NC+CAPE
Si-Foxo3+CAPE
Ferritin
GPX4
GAPDH
